# Integrating Modes of Transport in a Dynamic Modelling Approach to Evaluate Population Exposure to Ambient NO_2_ and PM_2.5_ Pollution in Urban Areas

**DOI:** 10.3390/ijerph17062099

**Published:** 2020-03-22

**Authors:** Martin Otto Paul Ramacher, Matthias Karl

**Affiliations:** Chemistry Transport Modeling, Helmholtz Zentrum Geesthacht, 21502 Geesthacht, Germany; matthias.karl@hzg.de

**Keywords:** transport emissions, nitrogen dioxides, particulate matter, urban air quality, chemistry transport modeling, population exposure, microenvironments

## Abstract

To evaluate the effectiveness of alternative policies and measures to reduce air pollution effects on urban citizen’s health, population exposure assessments are needed. Due to road traffic emissions being a major source of emissions and exposure in European cities, it is necessary to account for differentiated transport environments in population dynamics for exposure studies. In this study, we applied a modelling system to evaluate population exposure in the urban area of Hamburg in 2016. The modeling system consists of an urban-scale chemistry transport model to account for ambient air pollutant concentrations and a dynamic time-microenvironment-activity (TMA) approach, which accounts for population dynamics in different environments as well as for infiltration of outdoor to indoor air pollution. We integrated different modes of transport in the TMA approach to improve population exposure assessments in transport environments. The newly developed approach reports 12% more total exposure to NO_2_ and 19% more to PM_2.5_ compared with exposure estimates based on residential addresses. During the time people spend in different transport environments, the in-car environment contributes with 40% and 33% to the annual sum of exposure to NO_2_ and PM_2.5_, in the walking environment with 26% and 30%, in the cycling environment with 15% and 17% and other environments (buses, subway, suburban, and regional trains) with less than 10% respectively. The relative contribution of road traffic emissions to population exposure is highest in the in-car environment (57% for NO_2_ and 15% for PM_2.5_). Results for population-weighted exposure revealed exposure to PM_2.5_ concentrations above the WHO AQG limit value in the cycling environment. Uncertainties for the exposure contributions arising from emissions and infiltration from outdoor to indoor pollutant concentrations range from −12% to +7% for NO_2_ and PM_2.5_. The developed “dynamic transport approach” is integrated in a computationally efficient exposure model, which is generally applicable in European urban areas. The presented methodology is promoted for use in urban mobility planning, e.g., to investigate on policy-driven changes in modal split and their combined effect on emissions, population activity and population exposure.

## 1. Introduction

More than 60% of European citizens live in urban areas of over 10,000 inhabitants and even more in smaller urban environments [1]. Daily urban life circles around the same spaces and uses established mobility infrastructures to reach them. About 70% of transport-related air pollutants and 40% of road transport-related carbon dioxide (CO_2_) emissions arise from urban mobility [1]. In Europe, about 8% of the urban population in the EU-28 was exposed to levels above the EU annual limit value of 25 µg/m^3^ for fine particulate matter (PM_2.5_, particles smaller than 2.5 μm in aerodynamic diameter) and approximately 77% were exposed to concentrations exceeding the WHO air quality guideline (AQG) value of 10 µg/m^3^ for PM_2.5_ in 2017 [2]. Moreover, around 7% of the EU-28 urban population was exposed to nitrogen dioxide (NO_2_) concentrations above the annual EU limit value of 40 µg/m^3^ (which is equal to the WHO AQG limit value) [2]. Although these values have been decreasing since 2006, air pollution is still the most important environmental risk to human health in Europe [3].

Many epidemiological studies report statistical associations indicating that exposure to air pollution increases the risk of diseases such as lung cancer or chronic and acute respiratory and cardiovascular diseases, and premature deaths [4,5,6,7]. Although the relationship between NO_2_ and health effects is scientifically not as well founded as it is for PM_2.5_ [8,9,10,11], NO_2_ is often considered to be an indicator for other pollutants and the evidence for health effects related to NO_2_ exposure is rising in terms of physical [12,13,14,15,16,17] and psychological diseases [18]. 

Air pollution in urban areas is caused by different activities, such as power generation, industry, road transport, agriculture, shipping, and households. Cities are affected by the short-range and long-range transport of gaseous and particulate pollutants from the surrounding region; especially pollution levels of PM_2.5_ which in the urban background were shown to be largely controlled by atmospheric transport from up-wind regions [19]. In cities, road transport is one of the main contributors to urban air pollution. In 2017, the road transport sector was the most significant contributor to total nitrogen oxides (NO_x_) emissions (39%) and the second largest contributor of primary PM_2.5_ emissions (11%) in European cities [2]. However, the contribution of the road transport sector to population exposure to ambient concentrations in urban areas is considerably higher, because its emissions are closer to the ground.

Different measurement studies have shown that urban citizens commuting in cars and buses had the highest levels of air pollution exposure, followed by those commuting by a car with controlled ventilation settings, cyclists, and pedestrians [20,21]. Thus, reducing road transport emissions bears great potential to decrease negative impacts on health effects. Many cities’ regulatory bodies started to promote and support alternative modes of transport, such as car sharing, cycling, or walking and try to enhance public transport solutions. Moreover, several policies such as restricting vehicle access in designated areas or roads, low emission zones or calming traffic have been applied in European urban areas. To evaluate the impacts of other modes of transport and policies on urban citizens’ health effects, it is necessary to perform assessments on population exposure to air pollutants on the urban-scale, which can take into account different modes of transport.

Population exposure estimations are based on air concentration levels in the environments where people spend their time, and the amount of time they spend within them [22]. Thus, it is important to take into account both realistic pollution levels and population time–activity. 

Traditional approaches to account for pollutant concentrations in urban areas are usually based on data collected from fixed air quality monitoring stations and interpolation techniques to extend the spatiotemporal resolution for a given area and period [23]. Modern approaches often apply complex air quality models that take into account data on emissions, meteorology, and various physical and chemical processes to predict ambient concentrations of many types of pollutants at different spatial and temporal scales [21,24,25,26]. Although these models are connected with limitations such as model biases and inaccuracies of emissions data, it is possible to adjust these when monitoring data are available [27].

While modeling approaches to predict ambient air concentrations have been significantly improved, spatiotemporal information about population activity is still a major limitation in population exposure assessments [28]. Still, the most common approach in air quality exposure and health impact assessment studies is to consider residential addresses as a proxy of population location [29,30,31,32,33]. This approach tolerates differences between exposure at residential addresses and exposure that takes into account diurnal mobility behavior of individuals or a population [34,35,36,37,38]. In general, information regarding the diurnal spatial distribution of population is mostly derived from surveys, census data, or administrative records. Methods that are more recent apply GPS data [34,39,40,41], agent-based modeling (ABM) approaches [42,43,44], or different time-microenvironment-activity (TMA) models [25,26,45,46] to estimate mobility patterns. Nevertheless, each of these methods has its own shortcomings. Survey and census data are based on a limited number of samples while GPS data are rarely accessible and are connected with problems of data protection and privacy issues as well as with accessing the data [47]. Both methods provide individuals’ data that needs to be extrapolated to represent a population. This also holds true for the application of ABM, which is additionally computationally expensive, and thus mostly applicable to small areas and a limited set of individual activities, i.e., activity spaces and daily mobility [43]. The advantage of TMA models is that they enable a fast screening of dynamic populations in large areas, such as cities, regions, or countries. Moreover, TMA approaches for population exposure estimates can be applied in areas, where data on population activity are scarce [46], while most other approaches can only be transferred to other (urban) areas with considerable effort in data collection.

There is a need to develop innovative and operational approaches that combine ambient concentrations of air pollutants with mobility patterns and have the potential to improve exposure estimates for use in epidemiological studies. However, in urban areas where city-specific data on population activity are limited or not available, the modeling of citizen’s mobility patterns is a challenging task in estimates of population exposure. Our idea was to develop a TMA modeling approach which allows for the explicit consideration of spatiotemporal dynamics in the analysis of the collective exposures in different fine-granular modes of transport within large urban areas, with relatively low requirements on the availability of city-specific data.

This study presents a novel approach to analyze different modes of transport in dynamic population exposure studies based on a TMA model generically applicable to European urban areas. The new developments are based on OpenStreetMap data and allow for intra-urban exposure analysis on street-level resolution in European urban areas. The underlying data and assumptions are based on publicly available datasets and thus allow for applications in urban areas where specific data are missing. 

The new methodology was tested in the city of Hamburg (Germany), evaluating the population exposure to NO_2_ and PM_2.5_ during the year 2016 and comparing results with those calculated with a static (residential addresses-based) and a less detailed dynamic approach. The aim was to improve the analysis and evaluation of human exposure to urban air pollution risks by integrating different modes of transport in TMA approaches. The presented methodology is promoted for use in urban mobility planning, e.g., to investigate on policy driven changes in modal split and their combined effects on emissions, population activity, and population exposure.

This study utilizes an urban-scale chemistry transport modeling system in combination with a modified time-microenvironment-activity model to estimate population exposure to NO_2_ and PM_2.5_ in different transport environments. After describing the approach, models, and new developments, a case study is used to analyze air pollution and exposure. A limited sensitivity analysis is performed to examine impacts of key parameters on the calculated exposure. 

## 2. Materials and Methods

**Figure 1:**
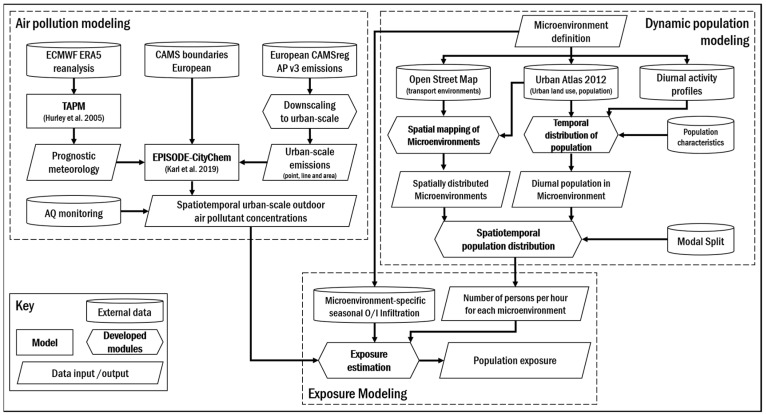
Schematic representation of the combined air pollution, dynamic population and exposure modelling approach.

To evaluate the urban population exposure to NO_2_ and PM_2.5_, a combined air pollution, dynamic population activity and exposure modelling approach was developed and applied (Figure 1). This approach is mainly based on the city-scale CTM EPISODE-CityChem [48] and a generic time-microenvironment-activity model to account for dynamic movements of the urban population within time and space in exposure estimations [46,49]. To investigate the impact of modal splits on exposure estimates, the generic TMA model was modified with new developments. The applied models, their setup and necessary input data, as well as modifications are explained in the following. The modeling approach was then applied to the Hamburg metropolitan area in the year 2016 to calculate the total population exposure and population-weighted exposure (PWE) to NO_2_ and PM_2.5_.

### 2.1. Modeling of NO_2_ and PM_2.5_ Concentrations in Hamburg for 2016

#### 2.1.1. Features of the EPISODE-CityChem Model

The urban-scale chemistry transport model EPISODE-CityChem [48] was applied to determine the NO_2_ and PM_2.5_ concentrations in the Hamburg urban area in the year 2016. EPISODE-CityChem combines a 3-D Eulerian grid model with sub-grid Gaussian dispersion models to resolve pollutant dispersion in proximity of point sources and lines sources. This approach allows for calculation of concentrations near pollution sources with high spatial resolution. Moreover, a simplified street canyon model (SSCM) is part of EPISODE-CityChem for better treatment of pollutant dispersion in street canyons in comparison with models without SSCM [48]. The SSCM module computes concentrations for the receptor points that are located in street canyons. The SSCM is based on the Open Street Pollution Model (OSPM) [50] but uses simplified street canyon geometry. Street canyons are approximated by three generic types for which average street canyon geometry properties are applied. A street canyon is identified for a line source if its geometric midpoint is located in a ground-level grid cell classified as urban land use. The street canyon model assumes that whenever wind blows over a rooftop in a street canyon, an hourly averaged recirculation vortex is formed inside the canyon. The concentration contribution of a line source to the concentration at a receptor is calculated as the sum of the direct contribution from the traffic plume and the contribution from the recirculation of the traffic plume due to the vortex.

EPISODE-CityChem solves the photochemistry of multiple reactive pollutants on the 3-D Eulerian grid. In order to use comprehensive chemical schemes in the urban air pollution model in a computationally efficient way, the number of compounds and reactions have been reduced to a minimum, while maintaining the essential aspects of urban atmospheric chemistry. The chemistry mechanism EmChem09-mod was applied which contains 70 gaseous compounds, 67 thermal reactions, and 25 photolysis reactions. The EmChem09-mod includes a large number of chemical interactions involving nitrogen oxides (NO_x_ = NO + NO_2_), ozone (O_3_), non-methane volatile organic compounds (NMVOC), sulfur dioxide (SO_2_), and other secondary pollutants that are important in the urban atmosphere. The chemistry scheme considers the oxidation of individual hydrocarbons by the hydroxyl radical (OH) and the nitrate radical (NO_3_).

Only a small portion of NO_x_ from motor vehicles and combustion sources are in the form of NO_2_, the main part being in the form of nitric oxide (NO). The largest fraction of ambient NO_2_ originates from the subsequent chemical oxidation of NO. In the sub-grid Gaussian models, the photo-stationary state (PSS) approximation for the reaction cycle of NO, NO_2_ and O_3_ is typically used. In EPISODE-CityChem, the PSS approximation is replaced by the compact reaction scheme EP10-Plume, which includes the formation of nitric acid (HNO_3_) and the photochemical degradation of formaldehyde (HCHO), an important constituent of vehicle exhausts, in addition to the reactions of NO, NO_2_, and O_3_. It has been shown that EP10-Plume gives very similar concentrations of NO, NO_2_, and O_3_ as the PSS close to roads [48]. Currently, PM_2.5_ is treated as a chemically inert tracer with no secondary particle formation. In the EPISODE-CityChem model, PM_2.5_ is removed from the atmosphere by dry deposition (diffusion, impaction, interception, and gravitational settling) and by wet scavenging.

#### 2.1.2. Model Configuration

EPISODE-CityChem reads meteorological fields to calculate dispersion parameters, vertical profile functions in the surface layer, and eddy diffusivities. Moreover, EPISODE-CityChem has the option to use the time-varying 3-D concentration field at the lateral and vertical as initial and boundary concentrations for selected chemical species. Emissions in EPISODE-CityChem can be treated as area sources (2-dimensional area of the size of a grid cell), line sources (line between two (x, y)-coordinates), and point sources (industrial and power plant stacks). 

EPISODE-CityChem predicts hourly concentrations of NO_2_ and PM_2.5_ on the 3-D Eulerian grid with a horizontal resolution of 1000 m in different vertical layers (first layer with a depth of 17.5 m). At the surface level, a regular receptor grid intercepts concentrations at 100 m resolution. The time-dependent surface concentrations of the pollutants at receptor points are calculated by summation of the Eulerian grid concentration of the corresponding grid cell (i.e., the background concentration) and the concentration contributions from the sub-grid models due to the near-source dispersion of line source and point source emissions. The hourly output of the 100 × 100 m^2^ surface receptor grid is used in this analysis to study impacts at ground level. In this study, an urban domain for Hamburg for a 30 km × 30 km area including most of the city was defined (Table 1).

Table 1 summarizes the model setup. The computational time for a 1-month simulation with EPISODE–CityChem was 9.8 h on an Intel^®^ Xeon ^®^ Platinum 8160 processor ((Dell Inc., Round Rock, TX, USA) at 2.1 GHz with 364 GB of RAM.

#### 2.1.3. Meteorological Setup of EPISODE-CityChem

In this study, prognostic meteorological fields from the meteorological component of the coupled meteorological and chemistry transport model TAPM (The Air Pollution Model, [51]) were applied. TAPM predicts three-dimensional meteorology based on an incompressible, non-hydrostatic, primitive equation model. In the meteorological module of TAPM, an urban scheme with seven urban land use classes [52] is used at the surface for better representation of urban parameters in the simulation of wind fields, e.g., by taking into account different anthropogenic heat fluxes or roughness lengths. In this study, three hourly synoptic scale ECMWF (European Centre for Medium-Range Weather Forecasts) ERA5 (European Reanalysis 5th generation) reanalysis ensemble means for 2016 on a longitude/latitude grid at 0.3 degree grid spacing have been used to drive the meteorological module of TAPM. Additionally, wind speed and wind direction from eight different meteorological stations (Supplement S2–T1) have been assimilated to nudge the TAPM simulations. Moreover, land cover classes and elevation have been updated with Corine Land Cover 2018 (CLC2018) raster data [53] with an original grid resolution of 100 m for better representation of the urban landscape. The meteorological fields simulated with TAPM have a horizontal resolution of 1 km × 1 km and a vertical resolution of 30 layers with different heights, following the EPISODE-CityChem vertical layer structure.

#### 2.1.4. Boundary Conditions

Background air concentrations are taken into account with hourly Copernicus Atmospheric Monitoring Services (CAMS) ensemble forecasts for carbon monoxide (CO), ammonia (NH_3_), NMVOC, NO, NO_2_, O_3_, peroxyl nitrates (PANS), particulate matter (PM_10_, PM_2.5_), and SO_2_. The CAMS regional ensemble is based on an ensemble of seven state-of-the-art numerical air quality models developed in Europe [54]. The output of the seven models is combined via an ensemble approach, in which the median value of the individual model outputs is considered. The spatial resolution of the regional forecast is 0.1 × 0.1 degrees for the whole of Europe, with nine vertical levels extending from the surface up to 500 hPa, and time resolution is one hour. The CAMS forecast concentrations were downloaded and interpolated to the horizontal and vertical resolution of the domain to be considered at the lateral and vertical borders of the urban domain.

#### 2.1.5. Urban Emissions

To account for all relevant emission sources in the study domain, emission data containing sector-specific and geo-referenced yearly emission totals are pre-processed with the EPISODE-CityChem interface for emission pre-processing, the Urban Emission Conversion Tool (UECT, [55]). UECT creates hourly varying emission input for point sources, line sources, and area source categories using sector specific temporal profiles and vertical profiles, based on annual totals of emissions. Temporal profiles from the SMOKE-EU model [56] are applied in UECT. For the lines source emissions, different diurnal profiles are applied for weekdays and weekends. The composition of the vehicle fleet assumes a fraction of 10% heavy duty and commercial vehicles. An NO_2_-to-NO_x_ ratio of 0.3 was applied to recalculate NO_2_ emissions for this study because of the expected higher real-world NO_2_ emissions from diesel vehicles. Total NMVOC emissions are distributed over individual VOCs of the chemical mechanism using the VOC split of the EMEP (European Monitoring and Evaluation Program) model [57] for all SNAP (Selected Nomenclature for sources of Air Pollution) sectors.

In this study, local annual emission totals of CO, NH_3_, NMVOC, NO_x_, PM_10_, PM_2.5_, SO_2,_ and methane (CH_4_) were applied for every sector with city-specific data or downscaled regional emission data if city-specific data were not available. These were applied as annual point, line, and area emission totals in UECT to produce hourly emissions for each emission category and afterwards applied in the EPISODE-CityChem setup for Hamburg in year 2016.

For point sources, industrial and commercial stack emissions for the year 2016 have been used, which have been reported to the environmental agency of Hamburg (Behörde für Umwelt und Energie) following the demands of the 11. BImSchV (Verordnung über Emissionserklärungen). Thus, there is a profound availability of data for point emission sources, which cover annual emissions and stack parameters, such as stack height, exit velocity and exit temperature. 

For line and area emissions, the CAMS regional anthropogenic emission inventory (CAMS-REG-AP) in version 3.1 for the year 2016 [58] was downscaled to the urban scale. The CAMS-REG-AP emission inventory provides sectoral annual emission totals on a 0.1° × 0.05° (lon × lat) for Europe. Sector specific proxies were applied to downscale the CAMS-REG-AP emission to a resolution of 1000 m × 1000 m. The downscaling procedure is described in detail in Supplement S1. 

#### 2.1.6. Road Transport Emissions

CAMS-REG-AP provides area-gridded emissions from road transport based on national emission reporting that take into account country-specific fleet compositions, fuel uses and state-of-the-art emission factors for each technology [58,59]. Nevertheless, for urban-scale CTM simulations with EPISODE-CityChem it is necessary to consider road traffic as line source to account for street canyon effects. The transformation of area sources for road transport to line sources requires spatial information on road networks as well as information on traffic density or annually averaged daily traffic volumes. The latter information was not available for the Hamburg urban area. Moreover, Kuik et al. 2018 [60] reported an averaged 50% underestimation of NO_x_ traffic emissions in urban core areas when downscaling regional emission inventories. Taking this into account, two more steps after downscaling road transport emissions from the CAMS-REG-AP inventory were added. First, all road emissions were multiplied with a factor of three in areas classified as urban an center following spatial information from the Global Human Settlement Urban Centre Database [61]. This factor follows the work of Kuik et al. 2018 [60] and remediates underestimations in regional emission inventories as well as other possible sources of model biases. 

Second, the modified road transport emissions were converted from gridded area sources into a dataset of line sources by applying major road types of the OpenStreetMap (OSM) database. Therefore, each 1 × 1 km^2^ grid cell of road traffic area emissions was separately intersected with downloaded OSM road links, which are tagged as motorway, trunk, primary, and secondary roads. The intersecting OSM road links’ lengths are used to calculate the total road length of all intersecting road links. The total road link length is used to derive a first weighting factor for each intersecting road link. A second weighting factor is derived based on the different road type of each road link intersecting the grid cell, to account for generic traffic densities of different road types, following the work of Ibarra-Espinosa et al. [62]. The combination of both weighting factors allows for top-down distribution of the grid cell emission value to all intersecting road lengths, taking into account length and road type. This is repeated for all grid cells of the road traffic area emissions grid. Thereby, all road traffic area emissions were distributed to OSM road links (line emission sources), which allows for consideration of SSCM in EPISODE-CityChem simulations.

### 2.2. Dynamic Population Modeling

Several studies have compared population exposure estimates based on static populations with spatially and temporally dynamic populations, emphasizing the need to account for population dynamics to reduce bias in population exposure estimates [21,23,24,25,26,36,37,38,39,45,46,63,64,65,66,67,68,69,70,71,72,73,74,75,76]. Thus, appropriate population exposure estimates in urban areas require the consideration of individual’s activities, which have a large degree of spatial and temporal dynamics. 

First, an overview of the application of a generic TMA methodology to account for dynamic movements of the urban population within time and space in exposure estimations [46] is presented. Second, newly developed modifications to the presented methodology that take into account the exposure in different modes of transport are introduced. The new developments allow for the combined and separate analysis of different modes of transport in urban-scale population exposure estimations embedded in a modelling approach, which is generically applicable to European urban areas. Thus, with the newly developed modifications, it is possible to investigate the impact of modal splits in urban-scale population exposure estimates. 

Following the national survey on mobility in Germany [42], the average modal split for the Hamburg urban area in 2017 was 27% by foot, 15% by bike, 36% by car (drivers and co-drivers) and 22% by public transport. The public transport split is 36% by bus, 32% by subway, 25% by suburban trains, 6% by regional trains, and 1% by ferryboats [43]. These figures are applied in the newly developed modifications to demonstrate the new implementations as well as to calculate the exposure in the city of Hamburg. Because of the low contribution to the modal split, we neglect public transport by ferryboats.

The TMA model and its modifications (UNDYNE: Urban Dynamic Exposure model) are written in the interpreted programming language R [77] and open-source distributed via GitHub. The model runs on different operating systems on single-core processors and needs a minimum of 8 Gigabyte RAM. Depending on the extent of the model domain and its resolution as well as the number of time steps, pollutants, and microenvironments calculated, the required computing time is about 1 h (1 year of hourly values for one pollutant in one microenvironment with a resolution of 100 × 100 m^2^).

#### 2.2.1. Microenvironment Mapping

A microenvironment is assumed to have homogeneous pollution concentrations patterns in time and space, e.g., at home, work or in transport [64]. In the following, the terms microenvironment and environment are used synonymously. Thus, human exposure will depend on the population time–activity pattern and concentration levels in the visited environments. In this study, three different approaches for the consideration of different environments were applied:

(1) *Static approach*: based on residential addresses and therefore consists of one microenvironment; the home environment.
(2) *Dynamic approach* [46]: consists of four different microenvironments, which are the home, work, other, and transport environment.
(3) *Dynamic transport approach*: newly developed modification of the *dynamic approach* to split the transport environment into seven different modes of transport.

In the following, the implementation of three approaches to determine population exposure in cities is briefly described.

The *static approach* is based on residential addresses, which are derived from the Urban Atlas 2012 dataset [78], given as land use and land cover classes (LULC) polygons that contain population estimates. The LULC polygons were rasterized to a population raster with a horizontal grid resolution of 100 m following the domain definition as applied in EPISODE-CityChem. This population raster is classified as home environment, because it is based on population counts at residential addresses.

**Figure 2:**
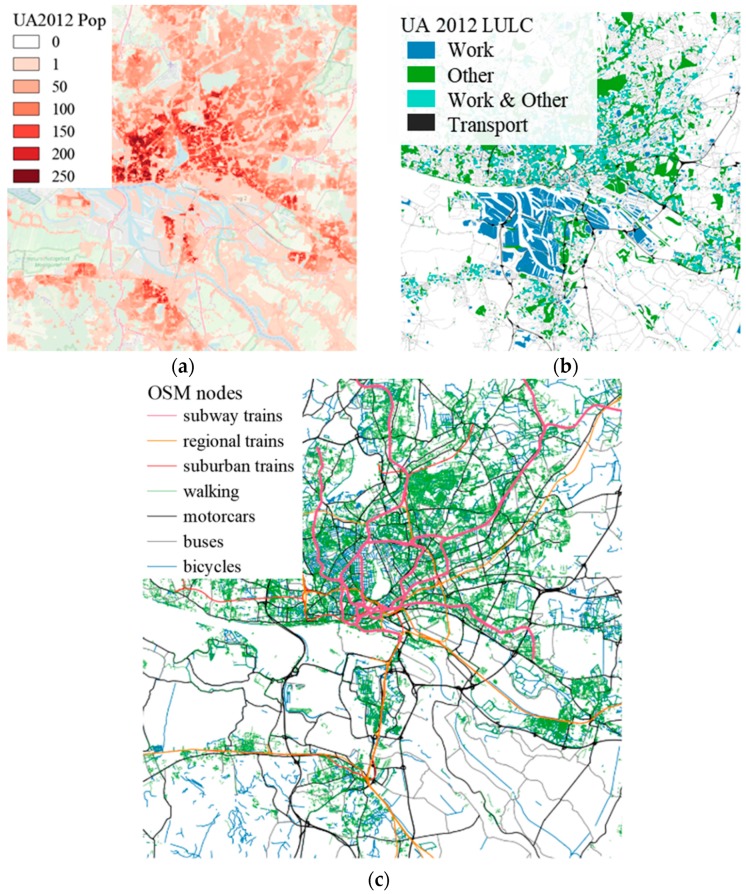
(**a**) Shows the population totals based on rasterized Urban Atlas 2012 (UA2012) land use and land cover classes (LULC) polygons and thus the spatial distribution of the home environment in all approaches. (**b**) Shows the spatial distribution of work, other and environments in both dynamic approaches and additionally the transport environment in the *dynamic approach* as mapped from the UA2012. (**c**) Shows environments for different modes of transport as derived from OSM data and applied in the *dynamic transport approach*.

The *dynamic approach* is based on a TMA methodology which has successfully been applied to the North European cities of Rostock, Riga, Gdansk, and Gdynia [46]. This methodology is based on mapping different microenvironments to LULC of the Copernicus Urban Atlas 2012 (UA2012) product [79]. This procedure allows for the definition of microenvironments in any European urban area, for which Urban Atlas data are available. Moreover, it is possible to consider the infiltration of outdoor air pollutant concentrations to indoor (micro-) environments in the exposure estimation. For this study, four different microenvironments were defined with the *dynamic approach* (home, work, other, and transport) and mapped to different LULC of the UA2012 (annex A1) for the Hamburg urban domain following Ramacher et al. [46] (Figure 2). The transport environment in the *dynamic approach* is based on LULC and is classified as “roads and associated land”, and therefore includes people travelling in the streets, bicycle lanes, and sidewalks. Nevertheless, there is no explicit distribution of modes of transport within the transport environment and railway transport modes are not included.

In the *dynamic transport approach*, the transport environment of the *dynamic approach* is split to take into account different modes of transport. This was mainly done by utilizing and translating OpenStreetMap (OSM) data (openstreetmap.org) to different transport environments, which represent different modes of transport. OSM is a collaborative project and contains free geographic data of the world, released with an open-content license. OSM databases are arranged by “keys” (categories of information) and “values” (particular information) to structure queries for accessing OSM data e.g., with an application programming interface (API). In this study, different transport environments, where people are moving through the city, i.e., in the routes of transportation, are of interest. In OSM, routes are ordered lists of nodes, representing a polyline, i.e., linear features such as streets, train lines or rivers. Based on seven different modes of transport, seven different queries to download OSM polylines with the R package “osmdata” [80] were created. The seven modes of transport are walking, cycling, going by car, bus, subway trains, suburban trains, and regional trains. These modes of transport are then represented by different transport environments respectively: walking, cycling, in-car, buses, subway trains, suburban trains, and regional trains (Figure 2). In the following, the transport environment definitions are used analogously for the modes transport. The walking environment mostly takes into account accessible footpaths, while the biking environment is a combination of designated cycle paths and roads that are accessible for bicycles. The in-car environment represents all modes of transport in motorized vehicles (except for buses) people are using to commute. Thus, the in-car environment is mainly consisted of the cities’ road network and highways, while the buses environment is only defined by major roads and does not take into account highways. The subway, suburban, and regional train environments are taking into account the corresponding rail networks. The “osmdata” queries containing keys and values for each mode of transport are listed in annex B. The downloaded OSM polyline features for each mode of transport are then rasterized using the “raster” package in R [81] resulting in raster grids that indicate the spatial coverage of a mode of transport in each grid cell.

In summary, all approaches share the same spatial distribution of home environments and the dynamic approaches additionally share the same spatial distribution of the environments work and other. However, while the *dynamic approach* only consists of one transport environment, the *dynamic transport approach* allows for detailed analysis of different modes of transport. Nevertheless, both dynamic approaches are offering the opportunity to introduce spatially and temporally dynamic populations in exposure assessments with general applicability to define microenvironments with high spatial resolution in European urban areas.

#### 2.2.2. Population Data and Diurnal Activities

While the definition and mapping of microenvironments provides the basis for spatial dynamics, application of population statistics and generic diurnal activity patterns provide the basis for temporal dynamics. In general, the principle idea is to calculate a total population figure for each microenvironment and hour of the day, which can then be spatially distributed to the corresponding microenvironment. In this study, population estimates for the year 2012 contained in the UA2012 dataset [78] were applied. The UA2012 LULC polygons were rasterized to a population raster with a horizontal grid resolution of 100 m following the domain definition as applied in EPISODE-CityChem. Moreover, the total population was increased by 4.3% to account for 2016 conditions, following reported population trends between 2012 and 2016 [82].

Due to the lack of more localized data of the population activity in Hamburg, the generic European diurnal activity profiles [46] were adopted. These profiles consist of different diurnal weekday and weekend patterns (Supplementary Material S3–1), which provide hourly varying percentages of the total population in the four different microenvironments home, work, road transport, and other. These diurnal activity patterns are reflecting a literature-based average of different European diurnal activity profiles [25,26,45,67,74,76] and have been applied as generic profiles in urban exposure estimations where activity patterns derived from surveys or GPS measurements have not been available [46].

Additionally, the average sum of 223,000 daily commuters during workdays in Hamburg [83] has been added to the total population and assigned to transport environments during rush hours and to the work environment during the day.

Following this approach, it is possible to calculate the total microenvironment-specific population for every hour of a diurnal cycle and spatially distribute it to each microenvironment. Thus, in combination with the mapping of microenvironments (Section 2.2.1) the presented methodology describes a dynamic TMA model.

### 2.3. Population Exposure Modeling

The principle idea of exposure is the combination of pollutant concentration values in the environments where people spend their time, and the amount of time they spend within them [4]. Thus, the introduced approaches to model urban-scale ambient pollutant concentrations and generic TMA models need to be combined to calculate meaningful exposure metrics [84], such as total population exposure or population-weighted population exposure (PWE).

In this study, only exposure to outdoor air pollution is considered; the effects of indoor air pollution sources are outside the scope of this study. Nevertheless, infiltration of ambient pollutant concentrations into indoor environments, such as the home, work, and the different transport environments, is considered. This is done by the application of microenvironment specific infiltration factors ($F_{inf}$) for different pollutant species, which can be defined as [26,67]:

(1) $$F_{Inf}=\frac{C_{ai}}{C_{a}}$$

where $C_{ai}$ is the indoor air concentration of a species originating from ambient air, and $C_{a}$ is the outdoor air concentration of species a. We gathered and aggregated $F_{inf}$ from the scientific literature for the different microenvironments and available seasons. While there is a lot of literature on experimental and modeling studies [85] to derive infiltration of PM_2.5_ outdoor to indoor concentrations [63,67,71,86,87,88,89,90,91], there are only a few studies on NO_2_ infiltration [45,87,92,93,94,95,96,97], especially when it comes to transport environments. Despite these limitations, it can be expected that the application of specific $F_{inf}$ for different modes of transport in combination with more differentiated transport environments as introduced in this study will provide valuable information.

Table 2 shows an overview of the applied $F_{inf}$ in this study, while Supplement S4 provides a full list of all reviewed $F_{inf}$.

The infiltration factors $F_{Inf}$ can then be considered specific for each microenvironment in the calculation of population exposure metrics. In general, exposure can be written as

(2) E=C×P×F

where E is the exposure, P is the population which is exposed to the ambient pollutant concentration C, both averaged over the same time, and F is the infiltration factor taking into account the outdoor to indoor infiltration, in case the population is indoors. This formula can be extended to calculate the time-averaged population exposure at a given location (or a grid cell) and for a given time period [24,26]:

(3) $$E_{i}=\overset{j_{max}}{\underset{j=1}{\sum }}F_{inf,\;j}\overset{t_{max}}{\underset{t=1}{\sum }}C_{i,t}\times P_{i,j,t}$$

where $E_{i}$ is the total exposure in all microenvironments of area i (e.g., a grid cell), $F_{inf,\;j}$ is the infiltration factor for microenvironment j, $C_{i,t}$ is the pollutant concentration in area i at time t, and $P_{i,j,t}$ is the number of people in area i attributed to microenvironment j at time t. Equation (3) can be defined for hourly, daily, or annual averages and allows for the modelling of exposure in one or various microenvironments, including peoples’ movements and the evaluation of outdoor pollution in indoor air. The total exposure value intends to assess general exposure levels since it does not take into account individual exposure patterns or an accumulated dose.

Another useful exposure metric is the population-weighted exposure, defined as average concentration of pollutants to which the population is exposed to in different environments:

(4) $$PWE_{i}=\frac{\sum_{j=1}^{j_{max}}F_{inf,\;j}\sum_{t=1}^{t_{max}}C_{i,t}\times P_{i,j,t}}{\sum_{j=1}^{j_{max}}\sum_{t=1}^{t_{max}}P_{i,j,t}}$$

where the denominator is the cumulative amount of the population within location *i* during a given period of time. $PWE_{i}$ can be calculated for single or multiple microenvironments per grid cell or as an average value for the total domain. 

In this study, we set numerical results on the population exposure, population-weighted exposure values as annual averages, as well as maps with a spatial resolution of 100 × 100 m^2^ of total exposure for the different approaches (*static*, *dynamic*, *dynamic transport*), and difference maps are presented.

## 3. Results 

### 3.1. Evaluation of Simulated NO_2_ and PM_2.5_ Concentrations

Simulated hourly results for NO_2_ and daily results for PM_2.5_ were compared to air quality data from the local air quality network at 16 monitoring stations measuring NO_2_ (hourly mean), and five monitoring stations measuring PM_2.5_ (daily mean). For the sake of brevity, diurnal, daily, and monthly variation and scatter plots of the modeled NO_2_ and PM_2.5_ concentrations are presented in Supplement S5. The diurnal profiles of modeled concentrations show bimodal distributions with two broad maxima due to increased urban traffic in the morning. The analysis was done with the R package “openair” [100].

Based on the evaluation, a modified set of road transport emissions was created to reduce the normalized mean bias (NMB) in modeled NO_2_ and PM_2.5_ pollutant concentrations for exposure calculation. Therefore, the annual totals of road transport emissions were multiplied with factors derived from the averaged maximum NMB of available traffic stations for NO_2_ (×1.3) and PM_2.5_ (×1.2). This modified set of road transport emissions was applied to the chemistry transport modeling system and the resulting pollutant concentrations are used as “reference” concentration in the exposure estimates.

In the statistical analysis of the model performance, the mean bias (MB), NMB, root mean square error (RMSE), Pearson correlation coefficient (r), index of agreement (IOA) and the fraction of predictions within a factor of two of observations (FAC2) to evaluate hourly NO_2_ (Table 3) and daily PM_2.5_ (Table 4) modeled versus measured concentration values (n) were evaluated. Definitions of statistical parameters can be found in annex C. 

All groups of stations for both pollutants and all measured vs. modeled values show FAC2 values of 0.57–0.8 for hourly NO_2_ and 0.78–0.88 for daily PM_2.5_, which satisfies the FAIRMODE acceptance criteria of FAC2 ≥ 0.3 for urban dispersion model evaluation [101,102].

The evaluation of statistical values for daily PM_2.5_ concentrations exhibits model performance with an average Pearson correlation coefficients of r = 0.5. The model tends to underestimate PM_2.5_ concentrations at urban stations (13ST, 20VE, 61WB) with an annual NMB of −15%. At the two traffic stations (64KS, 68HB), the model underestimates the measured concentrations with an annual NMB of −16%. 

When it comes to NO_2_, the model performs at urban and background stations with correlation coefficients of r = 0.35 to 0.58. The NO_2_ concentrations are underestimated at most urban and background stations, and in particular at the traffic stations.

The comparison of measured vs. modeled hourly NO_2_ concentrations and daily PM_2.5_ concentrations by type of station, did not reveal a stronger underestimation of modeled values at urban stations compared to background (only for NO_2_) stations. Thus, the general underestimation of modeled concentrations is probably a problem of underestimated emissions and/or boundary concentrations. 

### 3.2. Simulated NO_2_ and PM_2.5_ Concentrations and the Impact of Road Transport

Simulations with and without road traffic sources were performed to investigate the urban air quality in general and the contribution of road traffic in specific. The road traffic contribution to the modeled pollutant concentration was determined by performing zero-out simulations with EPISODE-CityChem, considering all emissions sources except road traffic emissions. The difference of simulations including all emission sources and simulations without road traffic emissions is the calculated contribution of road traffic emissions to pollutant concentrations.

**Figure 3:**
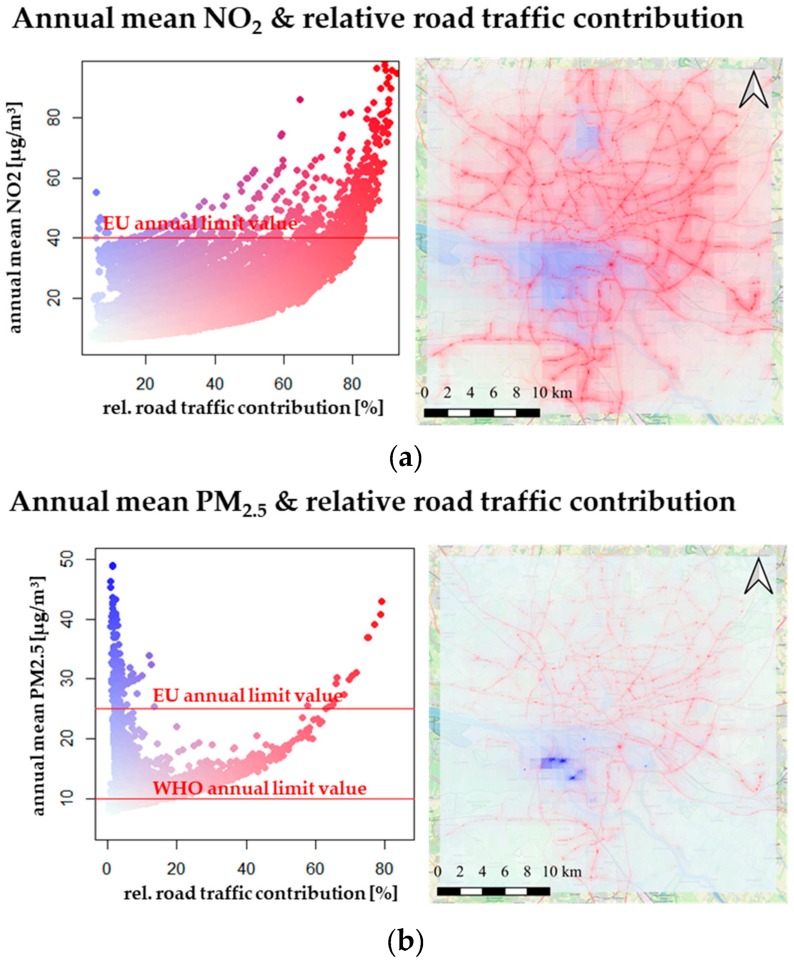
Modeled concentrations of annually averaged NO_2_ (**a**) and PM_2.5_ (**b**) plotted against relative contributions of road traffic. The range and color scale in the maps follows the colors of the scatter plot, which is the legend for the value maps’ value ranges. Darker red colored areas indicate higher concentrations with higher traffic contributions. Darker blue areas indicate higher concentrations and no traffic contributions. Concentrations increase with color intensity.

Annually averaged concentrations as an average of the entire urban domain are 15.4 µg/m^3^ NO_2_ and 9.7 µg/m^3^ PM_2.5_, with relative road traffic contributions of 33% and 5% respectively. Figure 3 shows the range of modeled concentrations of annually averaged NO_2_ and PM_2.5_, as well as relative contributions of road traffic as a scatter plot and a map.

For annually averaged NO_2_ concentrations, most exceedances of the EU limit value of 40 µg/m^3^ are connected to high contributions of road transport and thus are near roads. This correlates well with annually averaged NO_2_ concentrations measured at four traffic stations, because at all traffic stations the annual limit is exceeded too. There are only a few annual mean concentrations for NO_2_ above 40 µg/m^3^ predicted by the model for grid cells, which are not connected to high road transport contributions. Such exceedances occur mostly in the port area in which there is a lot of ship traffic and industry as well as close to the airport north of the city.

In terms of annually averaged PM_2.5_ values, the annual EU limit value of 25 µg/m^3^ show less modeled exceedances and there are only a few areas in which road traffic contributes to exceedances. Again, the industrial and port areas show elevated concentrations, with a few point sources (refineries, power plants) that are constantly emitting huge amounts of PM_2.5_ leading to high annually averaged concentrations.

### 3.3. Simulated Total Exposure to NO_2_ and PM_2.5_ Concentrations

Population exposure to ambient NO_2_ and PM_2.5_ concentrations was simulated by applying the *static approach*, the *dynamic approach*, and the *dynamic transport approach*. Thus, in the following, the results of each approach in terms of total exposure in different microenvironments and locations are presented and compared. Moreover, the PWE and contributions of road transport in all approaches and studied microenvironments are analyzed. This is followed by a discussion of uncertainties.

#### 3.3.1. Total Exposure in Different Approaches

**Figure 4:**
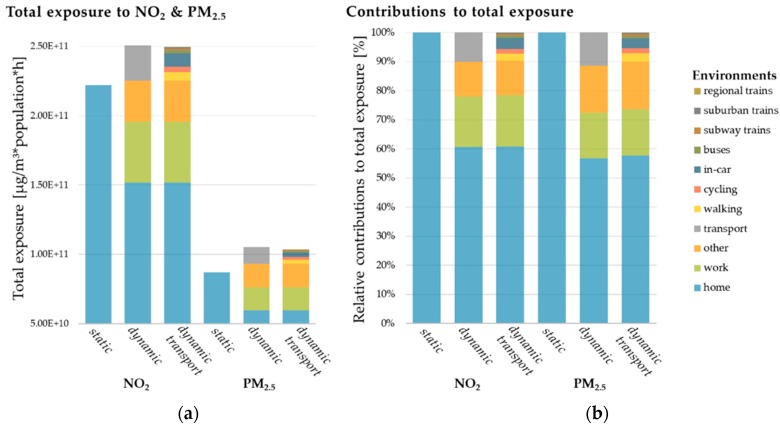
Annual total NO_2_ and PM_2.5_ exposure in Hamburg 2016 (**a**) and relative contributions of all environments to total exposures (**b**), calculated with three different approaches *static*, *dynamic*, *dynamic transport*.

The total population exposure to NO_2_ and PM_2.5_ in the Hamburg urban domain was computed for the year 2016, taking into account 8784 hourly values of concentration and population, as given in Equation (2). From now on, we will refer to that indicator as total exposure. The calculated sums of total exposure to NO_2_ and PM_2.5_ (Figure 4, Table S6-1) differ substantially, with the dynamic approaches giving a 13% higher total exposure for NO_2_ and 21% for PM_2.5_ than the *static approach*. The relative difference in total exposure between the two dynamic approaches is marginal for both pollutants (1% for NO_2_ and 2% for PM_2.5_). This is mostly due to the high contributions of the environments home, work, and other in the dynamic approaches (Figure 4). The population sojourning in the transport environment(s) in both dynamic approaches is affected by 10% of total NO_2_ and PM_2.5_ exposure. Thus, the total exposure is marginally affected by the OSM-based disaggregation of the transport environments and the consideration of a specific modal split in Hamburg in the *dynamic transport approach*. The similar total exposures calculated in the two dynamic approaches can be explained by the high relative contribution of in-vehicle exposure, compared to other modes of transport.

On the other hand, the modifications in the *dynamic transport approach* allow for evaluation of different transport modes. In terms of contributions of different modes of transport to the annual sum of exposure to NO_2_ in the transport environment of the *dynamic transport approach*, the in-car environment contributes with 40%, the walking environment with 26%, the cycling environment with 15%, and all other environments with less than 10% of the exposure in the transport environment, respectively. For PM_2.5_, the shares within the transport environment are similar, with 33% for the in-car environment, 30% for the walking environment, 17% for the cycling environment, and contributions of less than 10% for the other modes of transport.

#### 3.3.2. Differences in Spatial Distribution of Total Exposure

**Figure 5:**
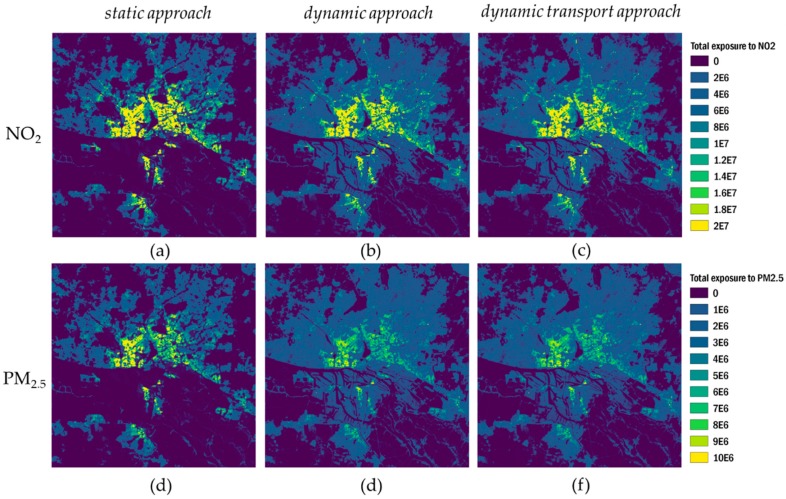
Annual total NO_2_ (**a**–**c**) and PM_2.5_ (**d**–**f**) exposures in Hamburg 2016, calculated with the *static approach* (**a**,**d**), the *dynamic approach* (**b**,**d**), and the *dynamic transport approach* (**c**,**f**).

**Figure 6:**
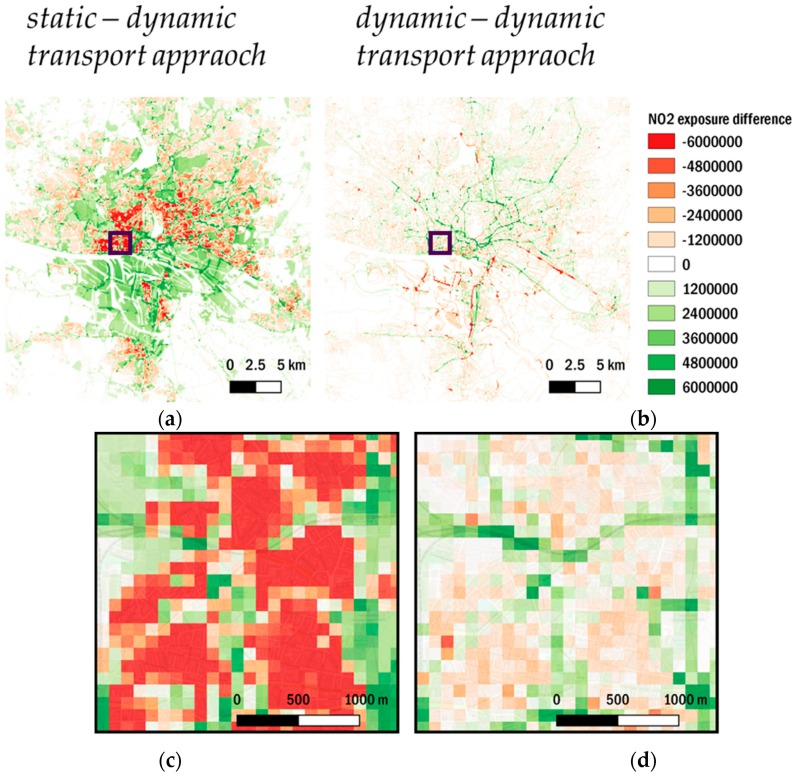
Differences in total exposure calculations for NO_2_ (**a**,**b**) in the total domain and zoomed (**c**,**d**) to the inner-city district of Hamburg-Altona (black frame in **a** and **b**) for the *dynamic transport approach* and the *static approach* (**a**,**c**), and the *dynamic transport approach* and the *dynamic approach* (**b**,**d**).

Figure 5 shows maps of total exposure to NO_2_ and PM_2.5_ for different approaches, while Figure 6 shows differences in total NO_2_ exposure for the *dynamic transport approach* compared to the *dynamic approach* and the *static approach*.

Comparing the different approaches in terms of spatial distribution, the high contribution of the home environment to total exposure is evident for all approaches. Moreover, the calculation of both, total exposure to NO_2_ and PM_2.5_, are strongly dependent on the number and location of people, thus the results in spatial distribution are similar for NO_2_ and PM_2.5_ total exposure. While in the *static approach* total exposure is only calculated for the home environment, its spatial pattern and the absolute high values of up to 2 × 10^7^ µg/m^3^ × pop total NO_2_ exposure (1 × 10^7^ µg/m^3^ × pop for PM_2.5_) are also visible in both dynamic approaches. Notably, the introduction of a dynamic population in the *dynamic approach* and the *dynamic transport approach* covers wider regions of the urban domain and considers them in the total exposure calculation. Due to the high exposure shares of the home, work, and other environments, the introduced spatiotemporal variability does not reveal changes in total exposure by integrating more transport environments in the *dynamic transport approach*, compared to the *dynamic approach* (Figure 5).

Thus, the differences of the *static approach* and the *dynamic approach* with the *dynamic transport approach* for NO_2_ were calculated and mapped in Figure 6. The differences for PM_2.5_ show similar spatial patterns and can be found in annex D. The comparison of the *dynamic transport approach* with the *static approach*, shows that there are substantial reductions of up to 5 × 10^6^ µg/m^3^ × pop total NO_2_ exposure in residential areas. On the other hand, total exposure increases in other areas of the urban domain such as the city center, industrial areas, the port area, highways and busy roads, or green urban areas. This demonstrates the impact of people “leaving their homes” to move to other environments. To isolate the effect of integrating more and different transport modes, the difference of the *dynamic transport approach* and the *dynamic approach* is analyzed. The difference shows mostly moderate reductions in areas surrounding the urban core whereas stronger reductions are found close to major roads. The finding reflects the different levels of detail in the applied land use classifications in both dynamic approaches, which are applied to the same number of people in the transport environment. While the number of people in the transport environment remains the same, their spatial distribution is changing. The transport environment in the *dynamic approach* is based on UA2012 LULC that solely take into account road networks and associated land, while the *dynamic transport approach* takes into account polylines that describe networks of possible routes through the city without associated land. Therefore, major reductions are mostly due to the differences in land use classifications. The associated land for road networks in the UA2012 LULC category consists mostly of non-accessible areas, while the applied OSM features are based on routes, which are accessible. Thus, the application of the *dynamic transport approach* leads to huge differences in areas that are highly affected by road traffic-related air pollution. The minor reductions in the surrounding area are again an effect of the UA2012 LULC for roads, which considers every road in the urban domain. The *dynamic transport approach* focuses on the most frequented road types (Table A1), not including every road in the urban domain. Thus, the minor reductions (Figure 6d) are due to a better representation of busy road networks and other modes of transport in the *dynamic transport approach*.

The increases in exposure by integrating different transport environments are mostly visible in densely populated areas in the center and south of the city (Figure 6). Moreover, the regional, suburban and subway train routes markedly contribute to total exposure in the *dynamic transport approach*. Besides the consideration of transport modes by rail which are distributed in the entire domain, the spatial pattern of changes through the introduction of new transport environments is generally showing an increase in total exposure in the city center and a reduction in the outskirts. Due to the fact that we are not taking into account densities of population activity in the domain but are equally distributing the same total population to each transport environment, we consider the increase of total exposure in areas close to the city center as an improvement to calculated total exposure.

**Figure 7:**
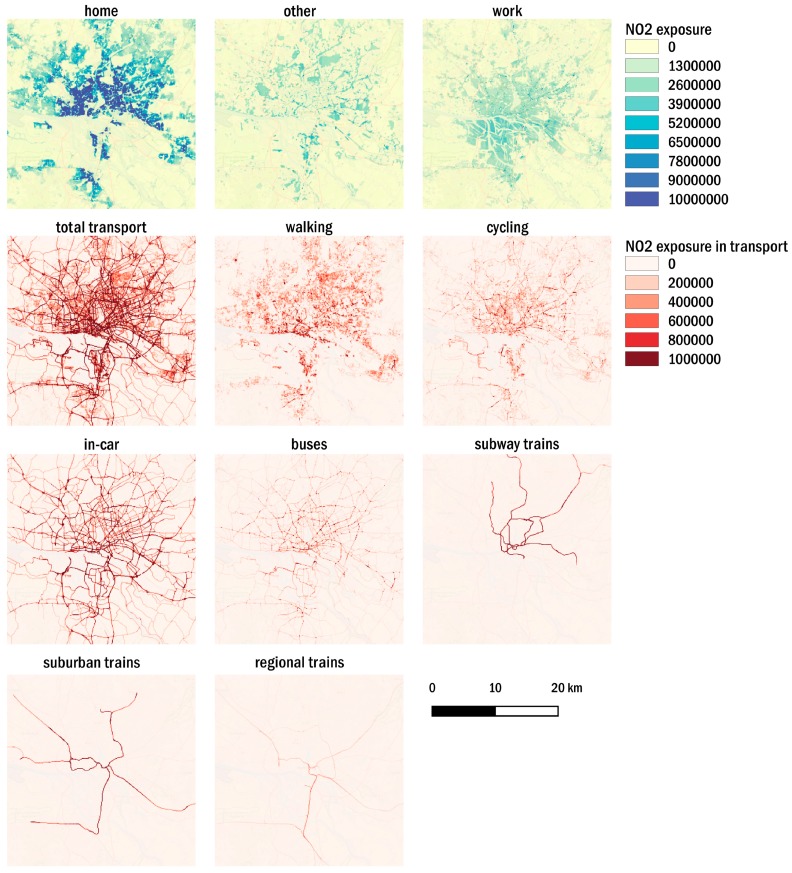
Total annual exposure to NO_2_ in all environments of the *dynamic transport approach* for Hamburg in year 2016.

The analysis by mode of transport (Figure 7, Figure S6-2) shows major impacts of population exposure in the in-car environment, which are similar for NO_2_ and PM_2.5_ in terms of spatial distributions. Besides the high share of people in Hamburg traveling by car (36%), high concentrations close to major roads due to road traffic is the main reason for the high contribution of the in-car environment to NO_2_ exposure (40%) and PM_2.5_ exposure (33%) in the transport environment. Overall, the in-car environment impacts total NO_2_ exposure by 4% and total PM_2.5_ exposure by 3%. Exposure hotspots in the walking environment are close to the port area at the northern side of the river Elbe close to the city center. In this area, pedestrians are exposed to high pollutant concentrations from road traffic, ships, and industry at the same time. Moreover, this area is a designated area for recreational and touristic activities, which might increase the total exposure in the walking environment when taking into account additional population by tourism. The total population exposure in the cycling environment is highest in the city center. The population exposure in the buses environment strongly depends on the routes and roads that buses are taking. In this study, we do not explicitly take into account bus routes as defined by the public transport operators, which will lead to uncertainty. However, the major road networks in the city and thus, most of the public transport operation network has been covered. The distribution of total exposure in transport modes by different train types is apparent in the *dynamic transport approach* with higher exposures in the subway trains environment compared to the suburban trains and regional trains environment. Nevertheless, the exposure to ambient concentrations inside train cars is strongly dependent on the infiltration factor. The same holds true for the in-car and buses environments. For this reason, sensitivities towards F_inf_ are discussed in Section 3.4.

#### 3.3.3. Impact of Road Traffic in Different Modes of Transport 

**Figure 8:**
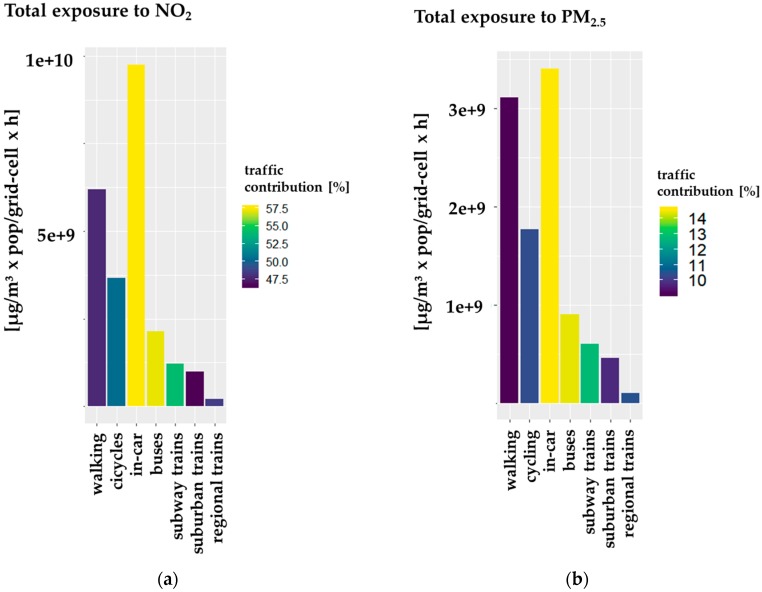
NO_2_ (**a**) and PM_2.5_ (**b**) total annual exposure and the relative contribution of road traffic in transport environments in the *dynamic transport approach* for Hamburg in year 2016.

The impact of road transport emissions to total exposure in different modes of transport of the *dynamic transport approach* (Figure 8) was calculated. For both NO_2_ and PM_2.5_ total exposure, the relative contribution is highest in the in-car environment with 58% for NO_2_ and 15% PM_2.5,_ respectively. In the buses environment, the impact is only slightly lower. The total exposure in the transport environment subway trains is influenced by 54% NO_2_ and 13% PM_2.5_ of road traffic emissions. The relative high contribution of NO_2_ in a transport mode, which is supposed to drive underground, is mostly due to the applied infiltration factors for the subway trains environment, which are discussed in detail in Section 3.4. Note that in Hamburg the subway lines are often not underground and follow dense traffic lanes, especially in the city center. For other railway modes of transport, the contributions to NO_2_ are 47% in suburban trains and 48% in regional trains, and for PM_2.5_ they are 10% and 11% respectively. The outdoor-only transport modes of walking and cycling are affected by road traffic emissions with 48% and 50% for total NO_2_ exposure, while the respective contributions to total PM_2.5_ exposure are 9% and 10%. Thus, there are substantial contributions to NO_2_ exposure by road traffic emissions in all transport environments, while the contribution to PM_2.5_ is much lower. However, the impact of road traffic as well as the total exposure to PM_2.5_ might be underestimated, as it mostly derives from regional contributions of PM_2.5_ to the urban background concentrations. In addition, there is no explicit consideration of particulate tire wear and road abrasion emissions in the road traffic emissions inventory, and no consideration of secondary particle formation in the applied chemical transport model (CTM).

Compared to the indoor environment home, the estimated road traffic contributions to NO_2_ and PM_2.5_ exposure are in average about 25% higher for NO_2_ and 40% higher for PM_2.5_. A relative contribution of 40% road traffic to total NO_2_ exposure in the home environment and up to 58% in the in-car environment was calculated. This fits well with the introduced 39% contribution by road traffic emissions as reported for European cities [2] and the hypothesis of higher road traffic contributions in environments where people are moving close to traffic emissions at the ground. The same qualitative result holds true for the reported 11% road traffic contribution to PM_2.5_ concentrations in European cities [2]; road traffic contributions of 8% in the indoor environment home and up to 15% in the in-car environment were calculated. Thus, the introduced approach introduces reasonable spatial variabilities in exposure estimations on the urban-scale.

### 3.4. Sensitivity of Ambient Concentration and Infiltration Factors in the Dynamic Transport Approach

Exposure estimates based on modeled pollutant concentrations and a TMA-modeled population are sensitive to a variety of parameters. Major uncertainties arise from applied emission inventories in chemistry transport modeling, specific infiltration factors for different indoor environments, as well as city-specific spatiotemporal distribution of populations in different microenvironments [46]. In the following, uncertainties connected to emissions as well as the infiltration of outdoor pollutant concentrations to indoor environments will be discussed.

In atmospheric chemistry transport model simulations, emission data are a key driver and a major source of uncertainty [103,104]. Modelling of emissions depends on the amount, temporal distribution, and spatial distribution of emissions and in some cases on meteorological conditions, all of which are connected with uncertainties. In this study, annual emission totals from a regional emission inventory for the year 2016 were spatially distributed to road-links and generic temporal profiles for road traffic activity following a top-down approach (Section 2.1.6). At the time of the study, the underlying emissions inventory CAMS-REG-APv3.1 only had the year 2016 as the most recent year available. Thus, the modeling system is dependent on more recent emission data, when applied to more recent years. Nevertheless, the emission inventory data by CAMS are planned to be updated for years that are more recent. Moreover, the modeled traffic emissions are not taking into account the variability due to changing traffic density, slowing down and idling of traffic, nor the effects of traffic congestion, which potentially increase emissions in streets, especially during rush hours [40].

To investigate uncertainties stemming from the simulated pollutant concentrations, simulated NO_2_ and PM_2.5_ concentrations were compared with measurements (Section 3.1). The results show the highest underestimations at measurement stations close to roads with an NMB of up to −35% for NO_2_ and −20% for PM_2.5_. To quantify the impact of this underestimation on uncertainty in exposure estimates, the reference emission scenario as defined in Section 3.1 was compared with the original lower traffic emissions inventory (minimum scenario). Additionally, a maximum emissions scenario was created by scaling the reference scenario with a factor derived from maximum calculated annual NMB, using scaling factors of 1.4 for NO_2_ and 1.3 for PM_2.5_ to the total road traffic emissions inventory. The minimum and maximum scenarios were applied in our modeling chain to identify minimum and maximum exposure compared to the reference exposure to NO_2_ and PM_2.5_ in all microenvironments. The results of the maximum and minimum emissions sensitivity runs (Figure S7-1) reveal relative changes in total exposure to NO_2_ of −9% and +6% for all approaches, and of −1% and +1% for PM_2.5_, respectively. In the transport environments of the *dynamic transport approach* there are uncertainties of −12% to +7% for NO_2_ and −2% to +1% for PM_2.5_. Thus, the calculated exposure values show in general a higher sensitivity to changing local NO_2_ emissions from road transport, while PM_2.5_ exposure is less sensitive.

A second major uncertainty in population exposure estimates is the impact of outdoor pollutant concentrations infiltrating indoor environments and the consideration of this by infiltration factors. Besides the fact that only ambient air pollutant concentrations are addressed and indoor sources and sinks of pollution such as tobacco smoking, cooking, heating, or cleaning are neglected, the application of F_inf_ is connected with a variety of assumptions that are influencing the total exposure estimates. In this study, infiltration factors were applied, which are specific for microenvironments of an area and are presuming a well-mixed and uniform distribution of pollutant concentrations in these microenvironments. Although these factors are based on indoor and outdoor measurements in specific microenvironments and areas of interest, they represent an average infiltration of, for example, the various buildings or car-cabins in the area. The variability of the infiltration factor is controlled by (i) the specific indoor infiltration of pollutants, depending on, for example, building structure, building stock, ventilation parameters, and behavior; and (ii) by the meteorological conditions, e.g., wind pressure and buoyancy effects. In our study, we applied an average F_inf_, which represents the infiltration of pollution as an average of the encountered variability in different environments. More sophisticated methods for modelling the infiltration of pollutants from outdoor to indoor environments [105,106] have become available recently that are able to represent such variability. Such approaches can take into account complex building structures, ventilation parameters and behavior, wind pressures, and buoyancy effects, surpassing the need to assume well-mixed pollutant concentrations. However, these approaches come with high computational costs as well as additional data needs in terms of building structure and ventilation parameters.

In terms of transport environments and specifically for different modes of transport, the availability of F_inf_ derived from experimental studies in car cabins and inside busses or trains is scarce for both PM_2.5_ and NO_2_. Thus, the applied F_inf_ for different transport environments in this study are critical parameters because they are based on only a small number of experimental studies, which might not reflect the conditions of our study area. While the in-car environment is highly sensitive to the infiltration of ambient concentrations into the vehicle-cabin, the transport modes of walking and cycling are not. Thus, depending on the ventilation behavior, the exposure might increase or decrease for the in-car environment. The same holds true for buses, subway trains, suburban trains, and regional train environments. Therefore, a sensitivity analysis was conducted taking into account a minimum and a maximum range of outdoor to indoor infiltration factors. The sensitivity of other indoor environments, such as home and work was investigated [46] and showed a linear relationship of infiltration factors and exposure estimates when keeping concentration levels constant. The same linear relationship is expected for transport environments in *dynamic transport approach*, due to the similar procedure of calculation. Nevertheless, a set of minimum and maximum F_inf_ for each transport environment was applied to estimate the range of uncertainty in terms of total exposure to NO_2_ and PM_2.5_ in comparison to the applied reference values (Table S7-1). 

The calculated impact of minimum and maximum F_inf_ in different environments showed linear decreases and increases in calculated exposure values (Figure S7-2). In terms of the impact on total exposure in the *dynamic transport approach,* the impact is +/− 1%. However, when analyzing each mode of transport separately, exposure results are highly sensitive towards F_inf_. The impact on exposure averaged over all modes of transport ranges between −14% and +8% for NO_2_ and between −14% and +13% for PM_2.5_. Moreover, the upper limit of the uncertainty range in the *dynamic transport approach* environments in-car and buses is exceeding the annual WHO AQG limit value of 10 µg/m^3^ for PM_2.5_. When analyzing the total exposure of all environments, the impact of F_inf_ for different modes of transport is low due to the relatively high contribution of other microenvironments on the total exposure. Nevertheless, in future studies it is desirable to apply more representative F_inf_ values for indoor and transport environments that are specific for different building infrastructures and different air-intake or ventilation techniques in buildings, car cabins or inside buses and trains. 

**Figure 9:**
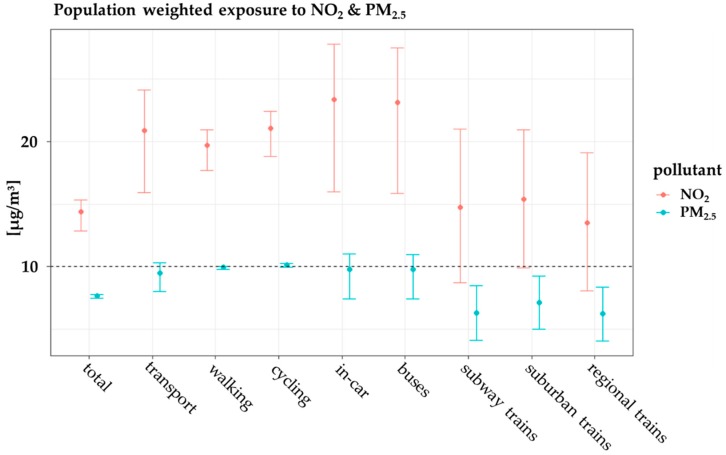
Population-weighted exposure (PWE) to NO_2_ and PM_2.5_ in the *dynamic transport approach* environments.

The separate sensitivity analysis of emissions and F_inf_ on exposure values showed a higher sensitivity for infiltration factors. To estimate the combined effect of both parameters, runs with the minimum emissions and the minimum F_inf_ scenario, as well as the maximum emissions and the maximum F_inf_ scenario were performed. Figure 9 shows the results of the combined simulation for both NO_2_ and PM_2.5_ in all transport environments of the *dynamic transport approach*.

There are higher uncertainties for exposure to NO_2_, with the highest ranges in the in-car, buses, and all train environments. In total, the transport environment in the *dynamic transport approach* shows an uncertainty range of −24% to +15%, while for PM_2.5_ the range is −16% to +14% (Table 5).

A third major uncertainty is the distribution of the total population to the specific microenvironments. The spatiotemporal distribution of population in this study is based on microenvironment definitions, which represent common urban environments in Europe, as well as diurnal activity patterns, which represent a European average activity profile based on available literature. To quantify and handle uncertainties that arise from spatiotemporal population distribution, city-specific data, e.g., based on surveys, mobile phone or GPS data, would be necessary. An investigation of those uncertainties is beyond the scope of the present study, as the base premise of the applied TMA modeling approach is the general applicability to any European urban area. 

Taking into account the analyzed uncertainties, we advocate for more transport-related experiments or modeling approaches on the infiltration of outdoor to indoor infiltration of air pollutants for exposure estimates.

### 3.5. Population-Weighted Exposure in Different Modes of Transport 

The population-weighted exposure (Equation (4)) shows the averaged pollutant concentrations representative for a specific microenvironment and thus the concentrations people are exposed to when visiting an environment. Therefore, the PWE can be used to analyze vulnerable microenvironments and areas where the population is exposed to critical concentrations of pollutants. With the newly developed *dynamic transport approach*, it is possible to calculate the population-weighted exposure for each environment separately (Figure 8). 

While the domain-wide averaged PWE to NO_2_ is showing lower concentrations of 14–15 µg/m^3^ for all non-transport-related environments in all approaches (Supplement S8), the transport-related microenvironments consist of values from 14–23 µg/m^3^ NO_2_ PWE (Figure 9). The *dynamic transport approach* reveals the highest PWE to NO_2_ in the in-car and buses environments (23 µ/m^3^). The walking and cycling environments are on average connected to NO_2_ PWE of 20–21 µg/m^3^, which is about 30% higher than staying at home. For PM_2.5_, the patterns are similar but with generally lower PWE. Indoor environments and total PWE in all approaches show modeled PWE to PM_2.5_ with 6–8 µg/m^3^, while it is highest for the cycling environment (10.17 µg/m^3^). Thus, moving by bike in the urban area is in average slightly exceeding the WHO AQG limit value for PM_2.5_. The second highest PWE in all transport modes is in the walking environment (9.94 µg/m^3^), followed by the environments buses (9.77 µg/m^3^) and in-car (8.16 µg/m^3^). These transport modes have a high chance to exceed the WHO AQG limit value for PM_2.5_ in modeled PWE values, due to their high sensitivity to different input parameters (Section 3.5), and the linear relationship of total exposure and PWE.

A comparison of the calculated PWEs with a review on the impact of different transport modes from measurements [20], shows the same trends for PWE to NO_2_. The in-car environment is followed by the transport environments buses, cycling, and walking. For PM_2.5_, we calculated a different order of importance, with the cycling and walking environment as environments with the highest average concentrations. However, different measurement studies on different pollutants showed different importance for transport environments in terms of PWE [89,107,108]. For the transport environment cycling and walking it has to be taken into account that these are representing active modes of transport and therefore inhaled doses will be higher. Nevertheless, consensus exists that despite the increased health risks associated with the higher inhaled dose of pollutants among active commuters rather than among commuters using motorized transport, the benefits of physical activity from active commuting are higher [20]. 

A comparison with reported PWEs for PM_2.5_ in urban areas in Germany for the year 2016 [109,110] shows higher reported values of 12.7 or 12 µg/m^3^ PM_2.5_ compared to the simulated values. This is probably due to underestimations in PM_2.5_ concentrations coming from the regional background as identified in the evaluation of simulated pollutant concentrations (Section 3.1). Nevertheless, the reported value holds to be valid for urban areas in Germany, while the simulated values with the *dynamic transport approach* are explicitly for Hamburg in the year 2016 and offer the opportunity to analyze PWE and total exposure in different microenvironments or for each 100 × 100 m^2^ grid cell of the study domain.

## 4. Conclusions

Air pollution is one of the greatest challenges facing cities today. In order to evaluate the effectiveness of policies and measures to reduce air pollution impacts on the urban-scale, in-depth population exposure assessments are needed. Due to road traffic emissions as the biggest source of NO_2_ exposure and second biggest source to PM_2.5_ exposure, it is even more important to account for a detailed transport environment in which people are moving through the city.

In this study, a methodology to estimate population dynamics based on generic activity profiles and publicly available land use data for microenvironment definitions was presented. This improves population exposure assessments in the transport environment. An approach for dynamic time-microenvironment activity including detailed transport environments is proposed in contrast to a dynamic approach disregarding detailed transport environment information as well as a traditional static approach.

The methodology proposed is applied to the city of Hamburg (Germany) for the year 2016 to evaluate population exposure to NO_2_ and PM_2.5_. Results show that, for spatially aggregated analysis at city level, the conventional static methodology calculates substantially lower total exposures to NO_2_ and PM_2.5_ when compared to applied dynamic approaches. The comparison of both dynamic approaches shows slightly lower total exposure values in the newly developed dynamic transport approach due to a better representation of people moving in different transport environments. 

Additionally the possibility and importance of explicit accounting for different modes of transport in urban population exposure estimates was demonstrated. In Hamburg, the in-car environment is the biggest contributor to total NO_2_ and PM_2.5_ exposure of all modes of transport, followed by walking and cycling. For both NO_2_ and PM_2.5_ total exposure, the relative contribution of road traffic emissions to population exposure is highest in the in-car and buses environments. 

Results of population-weighted exposure revealed exposure to PM_2.5_ concentrations above the WHO AQG limit value in the cycling environment. The results of the calculated PWE show good qualitative agreements, with measurement studies on exposure in transport environments. 

The performed sensitivity analysis showed high sensitivities to infiltration factors for in-cabin transport environments such as in-car, buses, and trains. The analysis of uncertainties revealed a high risk for exceeding the WHO AQG limit value for the transport environments walking, in-car, and buses.

The presented newly developed dynamic transport approach is integrated in a computationally efficient exposure model to calculate exposure estimates for different modes of transport and can easily be combined with transport mode specific inhalation rates to calculate health effects from air pollution in future studies. Taking into account the identified high exposure and PWE in the active transport modes walking and cycling, which are connected to higher inhalation rates, the detailed consideration of different modes of transport in urban exposure studies is a crucial foundation for reasonable health-effect studies. 

The developed dynamic transport approach is a novel method for urban planners to predict exposure hotspots and the impact of changing mobility patterns and mobility shifts on exposures to air pollutants, e.g., in the planning of new bicycle lanes in city centers in combination with the promotion of alternative modes of transport or policies on restricted access of motorized vehicles. A major advantage of this approach is the general applicability in European cities, without the need to obtain much city-specific data on population dynamics.

The study revealed current data gaps with respect to specific infiltration rates of pollutant concentrations from outdoor to indoor environments, especially in different transport environments such as in-car and train cabin environments. Moreover, we plan future studies to evaluate the results of the presented modeling approach, which is based on generic data for spatiotemporal population distribution, in conjunction with (individual) exposure measurement campaigns, agent-based modeling, and data on population dynamics that are more city-specific.

## Figures and Tables

**Table 1:** Overview of EPISODE-CityChem setup.

CTM Setup with EPISODE-CityChem	Setup for Hamburg 2016
Horizontal domain size (x × y)	30 × 30 km^2^
Horizontal domain resolution	1000 m
Model grid coordinate system	WGS1984 Universal Transverse Mercator (UTM) Zone 32N
Vertical dimension	Lowest Layer height 17.5 m 16 vertical layers below 1000 m Vertical top height 3750 m
Boundary Conditions	Hourly Copernicus Atmospheric Monitoring Services (CAMS) regional ensemble concentrations
Meteorology	Hourly meteorological fields simulated with The Air Pollution Model (TAPM), 1000 m horizontal grid resolution.
Point source emissions *	750 sources (federal emission reports, 11. BimSchV)
Line source emissions *	12625 road links (CAMS-REG-AP v3.1, OSM)
Area source emissions *	6430 sources, grid resolution 1000 m (CAMS-REG-AP v3.1)

* detailed description of emission inventories in Supplementary Material S4.

**Table 2:** Infiltration factors for PM_2.5_ and NO_2_ applied in this study. Values are derived from literature values as listed in the Tables S4-1 and S4-2.

Microenvironment	PM_2.5_		NO_2_		References
	Winter	Summer	Winter	Summer	
Residential	0.5	0.6	0.7	0.8	[26,45,67,71,92,93,96,97,98]
Work	0.5	0.6	0.75	0.85	[26,45,67,71,92,93,96,97,98]
Other	0.8	1	0.8	1	[26,46]
Transport	1	1	1	1	[25,71]
Walking, Cycling	1	1	1	1	-
In-car	0.7	0.8	0.9	0.9	[85,86,87,99]
Buses	0.9	0.9	0.9	0.9	[89]
Subway trains	0.7	0.7	0.6	0.6	[88,89,90]
Suburban trains	0.7	0.7	0.7	0.7	[88,89,90]
Regional trains	0.6	0.6	0.6	0.6	[88,89,90]

**Table 3:** Statistical performance of modeled versus measured hourly NO_2_ concentrations.

Site	*n*	FAC2	MB	NMB	RMSE	r	IOA
13ST	8687	0.69	−5.44	−0.20	16.11	0.49	0.52
17SM	8717	0.65	−18.26	−0.36	27.73	0.51	0.41
20VE	8680	0.77	−2.62	−0.07	19.28	0.44	0.47
21BI	8542	0.71	0.75	0.03	18.70	0.38	0.47
24FL	8599	0.68	−3.53	−0.16	15.00	0.50	0.54
51BF	8725	0.62	−4.68	−0.27	12.73	0.48	0.55
52NG	8684	0.60	−3.27	−0.22	12.48	0.42	0.52
54BL	8692	0.57	−6.32	−0.38	12.54	0.51	0.55
61WB	8682	0.71	3.22	0.12	18.71	0.35	0.42
64KS	8651	0.76	−9.66	−0.21	22.38	0.55	0.51
68HB	8675	0.63	−10.98	−0.18	37.41	0.46	0.50
70MB	8711	0.64	−19.96	−0.35	31.94	0.41	0.37
72FI	8721	0.66	2.45	0.12	16.57	0.42	0.48
73FW	8688	0.58	−0.57	−0.03	15.76	0.38	0.52
74BT	442	0.80	−3.91	−0.12	17.97	0.58	0.51
80KT	8686	0.79	2.47	0.08	17.65	0.44	0.50

**Table 4:** Statistical performance of modeled versus measured daily PM_2.5_ concentrations.

Site	*n*	FAC2	MB	NMB	RMSE	r	IOA
13ST	352	0.81	−3.03	−0.23	8.39	0.51	0.60
20VE	364	0.81	−1.62	−0.12	7.67	0.48	0.61
61WB	364	0.78	−1.18	−0.09	9.26	0.29	0.53
64KS	347	0.82	−2.49	−0.17	7.91	0.51	0.60
68HB	363	0.88	−2.27	−0.14	8.29	0.52	0.62

**Table 5:** Calculated sensitivities due to emissions and F_inf_ on total exposure and PWE to NO_2_ and PM_2.5_ calculations in the *dynamic transport approach*.

Transport Environment	NO_2_ Sensitivity		PM_2.5_ Sensitivity	
	Min	Max	Min	Max
walking	−10%	6%	−2%	1%
cycling	−11%	6%	−2%	1%
in-car	−32%	19%	−29%	28%
buses	−31%	19%	−24%	12%
subway trains	−41%	42%	−30%	30%
suburban trains	−36%	36%	−30%	30%
regional trains	−40%	41%	−35%	35%
transport	−24%	15%	−16%	+14%
total	−11%	6%	−3%	+2%

**Figure A1:**
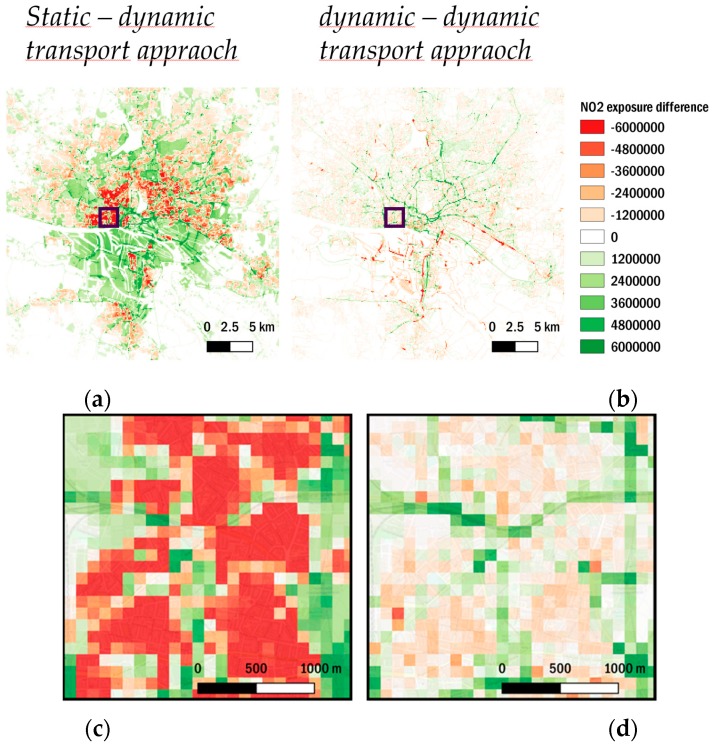
Differences in total exposure calculations for PM_2.5_ (**a**,**b**) in the total domain and zoomed (**c**,**d**) to the region of Hamburg-Altona (black frame in **a** and **b**) for the *dynamic transport approach* and the static approach (**a**,**c**) and the *dynamic transport approach* and the dynamic approach (**b**,**d**).

**Figure A2:**
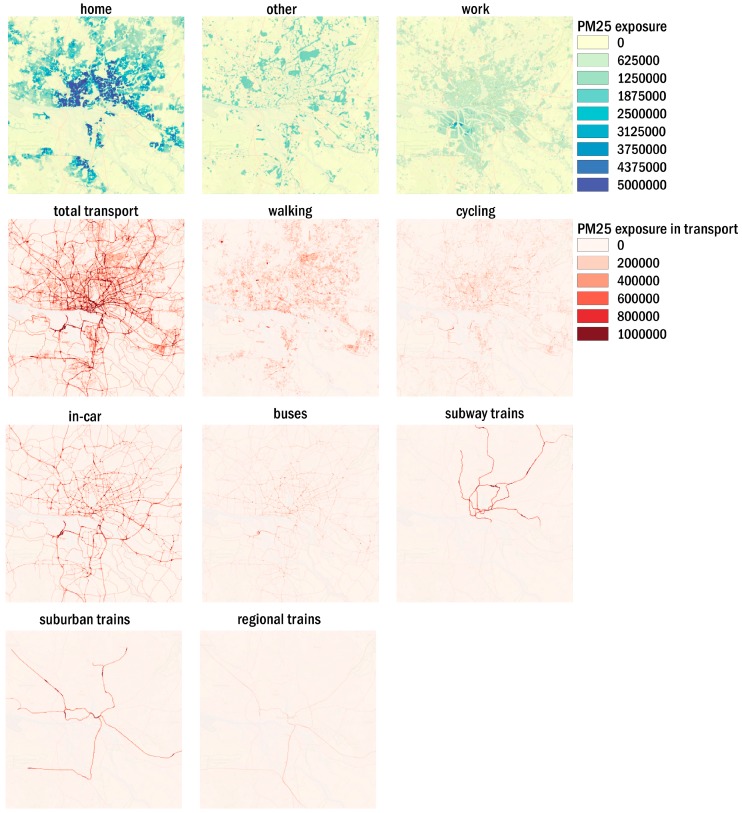
Total annual exposure to NO_2_ in all environments of the *dynamic transport approach* for Hamburg in year 2016.

## Supplementary Materials

The following are available online at https://www.mdpi.com/1660-4601/17/6/2099/s1, Supplement S1: Description of emission downscaling procedure, Table S1-1: Applied proxies to downscale regional emissions to the urban-scale, Table S2-1: Stations by the DWD used in wind field assimilation of TAPM runs, Table S2-2: Hamburger Luftmessnetz (HaLM) measurement stations, Figure S3-1: Diurnal activity profile for weekdays, Table S4-1: Infiltration ratios for PM_2.5_ in different microenvironments from literature, Figure S5-1: Hourly, monthly, and daily variation of modeled versus measured hourly NO_2_ concentrations, Figure S5-2: Hourly, monthly, and daily variation of hourly modeled versus daily measured PM_2.5_ concentrations, Figure S5-3: Scatter plots of measured vs. modeled (a) hourly NO_2_ and (b) daily PM_2.5_ concentrations, Table S6-1: Total calculated exposure to NO_2_ and PM_2.5_ of all approaches and in all microenvironments, Figure S6-1: Diurnal variation of hourly total exposure to NO_2_, Supplement S7: Sensitivity of modeled NO_2_ and PM_2.5_ exposure, Figure S7-1: Calculated PWE to NO_2_ and PM_2.5_ as well as uncertainties in all environments and approaches due to a minimum and a maximum road traffic emission scenario, Table S7-1: Applied infiltration factors for transport environments in the dynamic transport approach and their impacts on total exposure, Figure S7-2: Calculated PWE to NO_2_ and PM_2.5_ as well as uncertainties in all environments and approaches due to a minimum and a maximum road traffic emission scenario, Supplement S8: Population-weighted exposures, Figure S8-1: Simulated concentrations versus PWE grid cell values, Figure S8-2: Simulated concentrations and PWE grid cell values. The EPISODE-CityChem model is available at https://doi.org/10.5281/zenodo.3549415. The UNDYNE model for urban dynamic exposure calculations is available at https://doi.org/10.5281/zenodo.3687750. The simulated gridded hourly NO_2_ and PM_2.5_ concentrations, populations, and exposure for Hamburg in 2016 is available on request to M.R.

## Appendix A. Microenvironment Mapping in the *Dynamic Approach*

**Table A1:** Mapping of Urban Atlas 2012 LULC code with microenvironments.

Code	UA2012 Classification	Microenvironment
11100	Continuous Urban Fabric	Work (30%), Other (30%)
12100	Industrial, commercial, public, military, private	Work
13100	Mineral extraction and dump sites	Work
13300	Construction Sites	Work
12300	Port areas	Work
12210	Fast transit roads and associated land	Transport
12220	Other roads and associated land	Transport
14100	Green urban areas	Other
14200	Sports and leisure facilities	Other

## Appendix B. Microenvironment Mapping in the *Dynamic Transport Approach*

**Table A2:** Modes of transport and OpenStreetMap (OSM) queries for transport microenvironments definition.

Mode of Transport	OSM Key(s)	OSM Value(s)	Description
walking	“highway”	“footway”	For designated footpaths; i.e., mainly/exclusively for pedestrians.
Cycling (combination of three queries to cover all possible paths to ride a bike)	“highway”	“cycleway”	For designated cycle ways.
	“highway” “bicycle”	- “yes”	For roads which can be used by bikers.
	“cycleway”	-	Cycleway tagged on the main roadway or lane.
in-car	“highway”	“motorway”, “motorway_link", “trunk”, “trunk_link”, “primary”, “primary_link”, “secondary”, “secondary_link”, “tertiary”, “tertiary_link”	Restricted access, two lanes, freeways, Autobahn, most important roads that are not motorways, major, minor, residential roads. Additionally filtered by tunnels.
buses	"highway"	“primary”, “primary_link”, “secondary”, “secondary_link”, “tertiary”, “tertiary_link”	Major and minor urban road network.
subway trains	“railway”	“subway”	City passenger rail service, mostly underground.
suburban trains	“railway”	“light_rail”	higher-standard tram system.
regional trains	“railway” “usage”	“rail” “main”	passenger trains in the standard with heavy traffic.

## Appendix C. Statistical Indicators and Model Performance Indicators

In the statistical analysis of the model performance, the following statistical indicators are used: normalized mean bias (NMB), standard deviation (STD), root mean square error (RMSE), correlation coefficient (Corr), index of agreement (IOA), and the fraction of predictions within a factor of two of observations (FAC2). The overall bias captures the average deviations between the model and observed data and the NMB is given by:

(A1) $$NMB=\frac{\bar{M}-\bar{O}}{\bar{O}}$$

where $\bar{M}$ and $\bar{O}$ stand for the averaged model and observation results, respectively. The RMSE combines the magnitudes of the errors in predictions for various times into a single measure and is defined as

(A2) $$RMSE=\sqrt{\frac{1}{N}*\overset{N}{\underset{i=1}{\sum }}{\left(M_{i}-O_{i}\right)}^{2}}$$

where subscript *i* indicates the time step and *N* the number of observations. RMSE is a measure of accuracy, to compare prediction errors of different models for a particular data and not between datasets, as it is scale-dependent. The correlation coefficient (Pearson r) for the temporal correlation is defined as:

(A3) $$r=\frac{\sum_{i=1}^{n}\left(O_{i}-\bar{O}\right)\cdot \left(M_{i}-\bar{M}\right)}{\sqrt{\sum_{i=1}^{n}{\left(O_{i}-\bar{O}\right)}^{2}\cdot \sum_{i=1}^{n}{\left(M-\bar{M}\right)}^{2}}}$$

The index of agreement is defined as:

(A4) $$IOA=1-\frac{\sum_{i=1}^{N}{\left(O_{i}-M_{i}\right)}^{2}}{\sum_{i=1}^{N}{\left(\left|M_{i}-\bar{M}\right|+\left|O_{i}-\bar{O}\right|\right)}^{2}}$$

An IOA value close to 1 indicates agreement between modeled and observed data. The fraction of modeled values within a factor of two (FAC2) of the observed values are the fraction of model predictions that satisfy is defined as:

(A5) $$0.5\leq \frac{M_{i}}{O_{i}}\leq 2.0$$

For evaluation of modeled values in rural areas, the acceptance criteria is FAC2 ≥ 0.5, while in urban areas it is FAC2 ≥ 0.3 [101].

## Appendix E. List of Abbreviations

ABM	Agent-based modeling
API	Application programming interface
AQG	Air Quality Guideline
CAMS	Copernicus Atmospheric Monitoring Services
CH_4_	Methane
CLC2018	Corine Land Cover 2018
CO_2_	Carbon dioxide
CTM	Chemistry transport model
ECMWF	European Centre for Medium-Range Weather Forecasts
EMEP	European Monitoring and Evaluation Program
ERA5	European Reanalysis 5th generation
EU	European Union
F_inf_	Infiltration factor
GPS	Global positioning system
HCHO	Formaldehyde
HNO_3_	Nitric acid
IOA	Index of Agreement
LULC	Land use and land cover classes
MB	Mean bias
NMB	Normalized mean bias
NMVOC	non-methane volatile organic compounds
NO	Nitric oxide
NO_2_	Nitrogen dioxide
NO_3_	Nitrate radical
NO_x_	Nitrogen oxides
O_3_	Ozone
OH	Hydroxyl radical
OSM	OpenStreetMap
OSPM	Open Street Pollution Model
PANS	Peroxyl nitrates
PM_2.5_	particles smaller than 2.5 μm in aerodynamic diameter
PSS	Photo-stationary state
PWE	Population-weighted exposure
RMSE	Root mean square error
SMOKE-EU	Sparse Matrix Operator Kernel Emissions for Europe
SNAP	Selected Nomenclature for sources of Air Pollution
SO_2_	Sulfur dioxide
SSCM	Simplified street canyon model
TAPM	The Air Pollution Model
TMA	Time-microenvironment-activity
UA2012	Urban Atlas 2012
UECT	Urban Emission Conversion Tool
UNDYNE	Urban Dynamic Exposure Model
VOC	Volatile organic compound
WHO	World Health Organization
