# Supplementary material for: Integrating Modes of Transport in a Dynamic Modelling Approach to Evaluate Population Exposure to Ambient NO2 and PM2.5 Pollution in Urban Areas
Source: Int J Environ Res Public Health. 2020 Mar 22;17(6):2099. doi: 10.3390/ijerph17062099 (PMC7142857; doi:10.3390/ijerph17062099)
Supplement: Supplementary file 1 [file ijerph-17-02099-s001.pdf]

Supplementary materials

Integrating modes of transport in a dynamic modelling approach to evaluate urban population exposure to NO_2 and $PM_{2.5}$ pollution

Martin Otto Paul Ramacher[1,*], Matthias Karl [1]

[1] Chemistry Transport Modelling, Helmholtz Zentrum Geesthacht, 21502 Geesthacht, Germany; martin.ramacher@hzg.de (M.R.), matthias.karl@hzg.de (M.K.)

* Correspondence: martin.ramacher@hzg.de

Supplement S1—Emission downscaling procedure:

We used CAMS-REG-AP v3.1 (Granier et al. 2019) emission data downscaled the data from a horizontal resolution of ca. a 0.1° x 0.05° (lon × lat) to a ca. 1 km x 1 km resolution. CAMS-REG-AP v3.1 (Granier et al. 2019) follows the sector classification GNFR, which is an aggregated version of the NFR (Nomenclature For Reporting, (EEA 2016)) and provides sectoral annual emission totals for Europe. We applied sector specific proxies to downscale the regional emission inventories to a resolution of 1 × 1 km^2. The downscaling procedure followed a generalized framework:

1. First, a set of suitable proxies for the sector of interest was defined.

2. Second, the proxy grids were gridded and resampled (nearest neighbor) to match extent and resolution of the EPISODE-CityChem modeling domain. We applied proxy data from different sources, such as Corine Land Cover 2018 (CLC2018, (Copernicus Land Monitoring Service 2018)), the Global Human Settlement Population Grid (GHS-Pop, (Florczyk et al. 2019)), and source information of the European Pollutant Release and Transfer Register (E-PRTR). A full list of applied proxies can be found in Supplement Table S-T2.

3. Third, the chosen proxy was normalized by calculating a factor that is indicating the proportion of each proxy data type in one high-resolution grid cell within one coarse grid cell.

4. In a fourth and last step, these factors are used to downscale the respective emissions in the respective area top-down. Thus, the spatial distribution information of the coarse CAMS-REG-AP grid is considered in downscaling to the high-resolution grid (1 km x 1km). This framework was applied to downscale all gridded sector emissions of the CAMS-REG-AP emission inventory to create are emissions for the urban domain.

Table S1-1. Applied proxies to downscale regional emissions to the urban-scale.

Type	GNFR	SNAP	Proxy / Downscaling Approach	Emission Data Source
Point	A_PublicPower	1	If reported emissions are lower than CAMS-REG-AP v3.1: residual emissions are distributed to SNAP 1, 3 or 4 area emissions (see below)	11. BImSchV & CAMS-REG-APv3.1
Point	B_Industry	3, 4		11. BImSchV & CAMS-REG-APv3.1
Line	F_RoadTransport	7	Downscaled with GHS-Pop, weighted with factor of 3 (Kuik et al. 2018 (Kuik et al. 2018)) in areas defined as urban center by GHS Urban Centre database and distributed to major Open Street Map road types.	CAMS-REG-APv3.1
Area	A_PublicPower	1	CLC2018 code 121	CAMS-REG-APv3.1
Area	B_Industry	3, 4	CLC2018 code 121	CAMS-REG-APv3.1
Area	C_OtherStationaryComb	2	GHS-Pop	CAMS-REG-APv3.1
Area	D_Fugitives	5	CLC2018 code 121	CAMS-REG-APv3.1
Area	E_Solvents	6	GHS-Pop	CAMS-REG-APv3.1
Area	G_Shipping	8	CLC2018 codes 123, 523, 522	CAMS-REG-APv3.1
Area	H_Aviation	11	CLC2018 codes 124	CAMS-REG-APv3.1
Area	I_Offrad	11	CLC2018 codes 121, 211, 212, 213, 221, 222, 223, 231, 241, 242, 243, 244, 123, 124, 521, 522,523, 131, 132, 133	CAMS-REG-APv3.1
Area	J_Waste	9	E-PRTR information combined with CLC2018 polygons.	CAMS-REG-APv3.1
Area	K_AgriLiveStock	10	CLC2018 codes 211, 212, 213, 221, 222, 223, 231, 241, 242, 243, 244	CAMS-REG-APv3.1
Area	L_AgriOther	10		CAMS-REG-APv3.1

Supplement S2—Meteorological and Air Quality monitoring stations

Table S2-1. Stations by the DWD used in wind field assimilation of TAPM runs.

DWD Station Code	UTM Zone 32N Easting	UTM Zone 32N Northing
DWD_Hamburg	565337	5943161
DWD_Hannover	546054	5812908
DWD_Bremen	486451	5877295
DWD_Cuxhaven	480655	5969242
DWD_Kiel	582485	6039856
DWD_Schwerin	657803	5946395
DWD_Luechow	643524	5871337
DWD_Hamburg	565337	5943161

Table S2-2. Hamburger Luftmessnetz (HaLM) measurement stations with type of stations and measured pollutants.

Station Code	Station Name	Station Type	Altitude	Lon	Lat	Pollutants Measured
13ST	Sternschanze	Urban	3.5	9.96834	53.56449	NO_2, O_3, PM_{10}, $PM_{2.5}$, SO_2
17SM	Stresemannstrasse	Traffic	1.5	9.957387	53.56086	NO_2, O_3, PM_{10}
20VE	Veddel	Urban	3.5	10.02197	53.52291	NO_2, PM_{10}, $PM_{2.5}$, SO_2
21BI	Billbrook	Urban	3.5	10.08214	53.52943	NO_2, PM_{10}, SO_2
27TA	Tatenberg	Urban	3.5	10.08365	53.48814	NO_2, O_3
51BF	Bramfeld	Urban	3.5	10.1105	53.63089	NO_2, O_3
54BL	Blankenese	Background	3.5	9.786191	53.56806	NO_2, O_3, SO_2
52NG	Neugraben	Background	3.5	9.857199	53.48098	NO_2, O_3
61WB	Wilhelmsburg	Urban	3.5	9.990543	53.50792	NO_2, PM_{10}, $PM_{2.5}$, SO_2
64KS	Kielerstrasse	Traffic	1.5	9.944616	53.56437	NO_2, $PM_{2.5}$
68HB	Habichtstrasse	Traffic	1.5	10.05372	53.59235	NO_2, PM_{10}
70MB	MaxBrauerAllee	Traffic	1.5	9.943065	53.55573	NO_2, PM_{10}
72FI	Finkenwerder West	Urban / Industry	3.5	9.844191	53.53622	NO_2
73FW	Finkenwerder Airbus	Urban / Industry	3.5	9.831633	53.53136	NO_2, PM_{10}
74BT	Billstedt	Urban	3.5	10.10286	53.53851	NO_2, PM_{10}
80KT	AltonaElbhang	Urban	3.5	9.944915	53.54524	NO_2, O_3, PM_{10}, SO_2

Supplement S3—Diurnal activity profile for weekdays

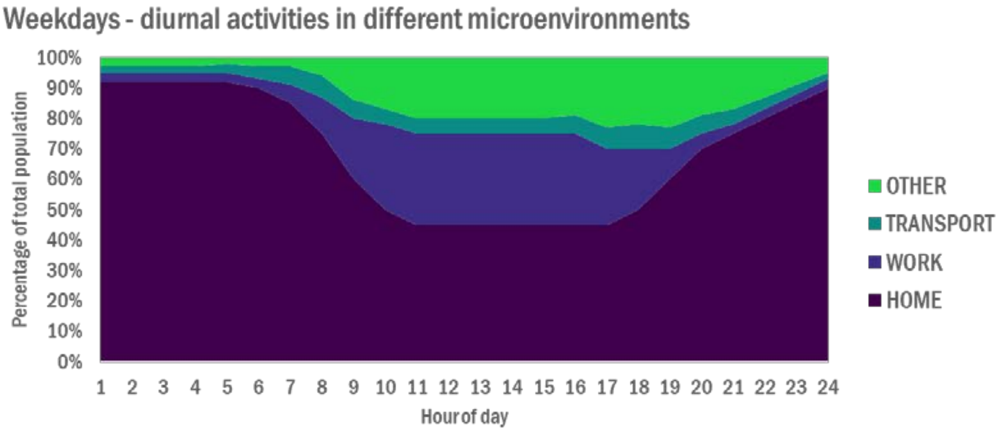

Figure 3. Diurnal activity profile for weekdays as derived from (Ramacher et al. 2019..)

Supplement S4—Indoor infiltration factors for different microenvironments

While there is a solid foundation for experimentally derived infiltration factors for PM2.5 in almost all microenvironments and modes of transport (S4-1), there are major knowledge gaps in terms of infiltration factors for NO2 (S4-2), especially in terms of different modes of transport. Thus, we made reasonable assumptions for missing values based on existing literature values. To identify the uncertainty that arises from the chosen infiltration factors for different modes of transport, we performed a sensitivity analysis in chapter 3.4 in the manuscript.

Table S4-1. Infiltration ratios for $PM_{2.5}$ in different microenvironments from literature

Microenvironment	Min	Mean	Max	Location	Season	Reference
Transport	-	1	-	London	annual	Smith et al. (2016), Singh et al. (2020)
Car	0.2	-	0.9	Hangzhou	winter	Tong et al. (2019)
Car	0.6	0.92	1	Los Angeles	spring	Fujita et al. (2014)
Car	0.3	-	0.98	North Raleigh	summer	Jiao and Frey (2013)
Car	0.43	-	0.99	NA	NA	Liu and Frey (2011)
Bus	-	0.91	-	Nanjing	annual	Shen and Gao (2019)
Subway		0.37		Beijing	spring	Jia et al. (2018)
Subway	0.56	-	0.66	Hongkong	winter	Li et al. (2018)
Subway	-	0.94	-	Taipei	NA	Shen and Gao (2019)
Subway	-	0.81	-	Seoul	NA	Shen and Gao (2019)
Subway	-	0.82	-	Los Angeles	NA	Shen and Gao (2019)
Subway	-	0.18	-	Naples	NA	Shen and Gao (2019)
Subway	-	0.79	-	Singapore	NA	Shen and Gao (2019)
Buildings	0.37	0.57	0.7	Helsinki	annual	Soares et al. (2014)
Residential	0.35	0.56	0.86	London	annual	Smith et al. (2016)
Residential	0.42	0.59	0.76	Europe	annual	Hänninen et al. (2004)
Work	0.23	0.47	0.71	Europe	annual	Hänninen et al. (2004)
Residential	-	0.7	-	Athens. Greece	Winter	Hänninen et al. 2011
Residential	-	0.63	-	Basle. Switzerland	Winter	Hänninen et al. 2011
Residential	-	0.59	-	Helsinki. Finland	Winter	Hänninen et al. 2011
Residential	-	0.61	-	Prague. Czech	Summer	Hänninen et al. 2011
Residential	-	0.53	-	Florence	Winter	Hänninen et al. 2011
Residential	-	0.7	-	Riverside. USA	-	Ozkaynak et al. (1993)*
Residential	-	0.56	-	Riverside. USA	-	Ozkaynak et al. (1993)*
Residential	-	0.62	-	Chongju. Korea	-	Lee et al. (1997)*
Residential	-	0.66	-	Birmingham. USA	-	Lachenmyer and Hidy (2000)*
Residential	-	0.35	-	Baltimore. USA	-	Landis et al. (2001)*
Residential	-	0.7	-	Boston, USA	-	Long et al. (2001)*

Residential	-	0.48	-	Seven cities. USA	-	Wallace et al. (2003)*
Residential	-	0.45	-	North Carolina	-	Williams et al. (2003)*
Residential	-	0.51	-	Three cities. USA	-	Reff et al. (2005)*
Residential	-	0.55	-	North Carolina. USA	-	Wallace and Williams (2005)*
Residential	-	0.48	-	L.A. USA	-	Sarnat et al. (2006)*
Residential	-	0.51	-	Houston, USA	-	Meng et al. (2007)*
Residential	-	0.66	-	L.A. County, USA	-	Meng et al. (2007)*
Residential	-	0.65	-	Elizabeth, USA	-	Meng et al. (2007)*
Residential	-	0.51	-	Helsinki, Finland	-	Hoek et al. (2008)*
Residential	-	0.3	-	Athens, Greece	-	Hoek et al. (2008)*
Residential	-	0.38	-	Amsterdam, NL	-	Hoek et al. (2008)*
Residential	-	0.37	-	Birmingham, UK	-	Hoek et al. (2008)*
Residential	-	0.63	-	Three Cities, USA	-	Meng et al. (2009)*
Residential	-	0.72	-	Three Cities, USA	-	Meng et al. (2009)*

* Adapted from Chen and Zhao (20.11)

Table S4-2: Infiltration ratios for $PM_{2.5}$ in different microenvironments from literature

Microenvironment	Min	Mean	Max	Location	Season	Reference
Car	0.91	0.99	1	Los Angeles	spring	Fujita et al. (2014)
Building	0.38	-	0.45	Los Angeles	winter	Hazlehurst et al. (2018)
Building	0.27	-	0.53	Los Angeles	summer	Hazlehurst et al. (2018)
Building	-	0.76	-	Europe	annual	Kousa et al. (2002)
Building	0.11	0.31	0.59	London	annual	Smith et al. (2016)
Building	0.3	0.545	0.66	Switzerland	annual	Meier et al. (2015)
Building	0.88	-	1	France	annual	Blondeau et al. (2005)
School	-	0.9	-	average	annual	Salonen et al. (2019)
Office	-	0.9	-	average	annual	Salonen et al. (2019)
Buildings	-	0.8	-	Seoul	winter	Bae et al. (2004)
Buildings	0.73	0.8	0.9	UK	annual	Challoner and Gill (2014)

Supplement S5—Evaluation of modeled NO_2 and $PM_{2.5}$ concentrations.

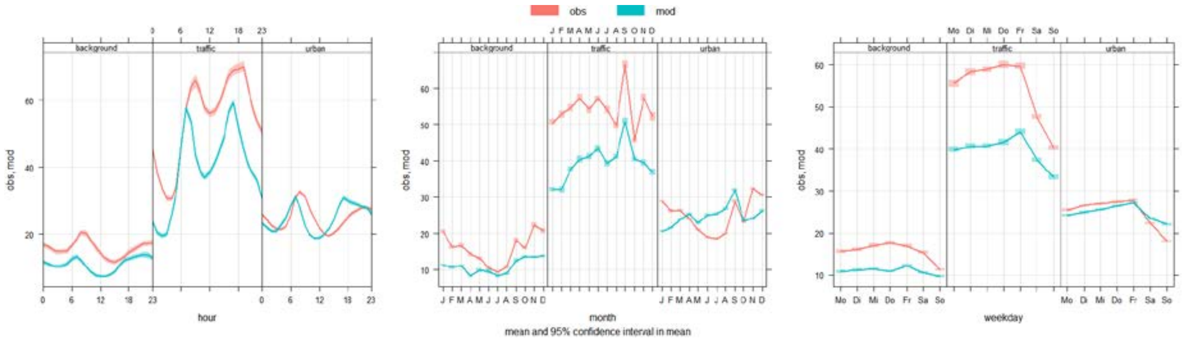

Figure S5-1: Hourly, monthly and daily variation of modelled versus measured hourly NO_2 concentrations averaged by background, traffic and urban stations.

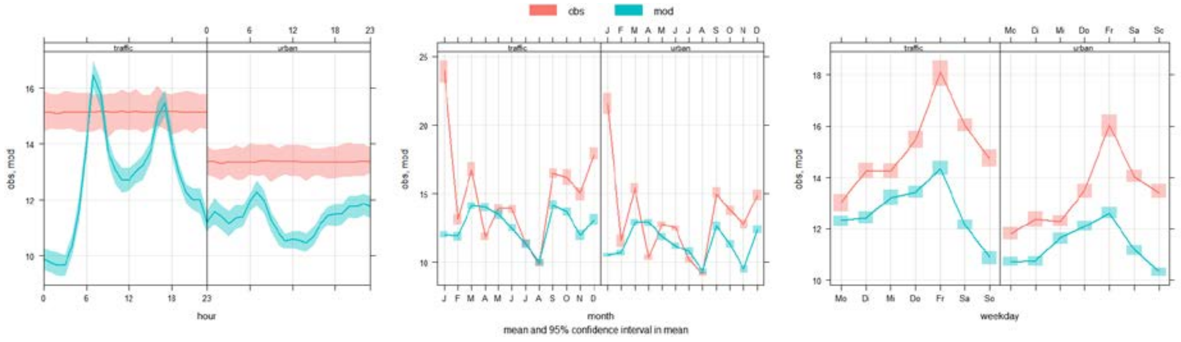

Figure S5-2: Hourly, monthly and daily variation of hourly modelled versus daily measured $PM_{2.5}$ concentrations averaged by traffic and urban stations.

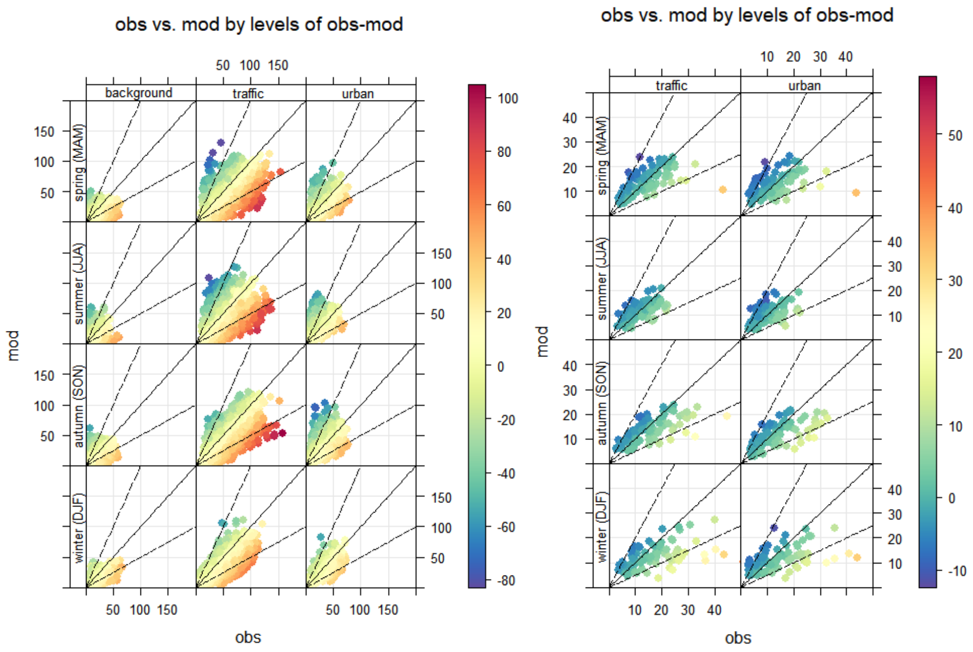

(a) (b)

Figure S5-3: Scatter plots of measured vs. modelled (**a**) hourly NO_2 and (**b**) daily $PM_{2.5}$ concentrations by type of station (urban background, traffic, urban) and season (from top to bottom: spring, summer, autumn, winter), showing the difference of measurement and model color coded.

Supplement S6 – Modeled NO2 and PM2.5 exposure

Table S6-1: Total calculated exposure to NO2 and PM2.5 of all approaches and in all microenvironments. Additionally the relative contribution of microenvironments in the *dynamic transport approach* is shown.

	**Total Exposure**			**relative contributions of *dynamic transport approach* environments to**	
	Static approach	***Dynamic approach***	***Dynamic transport approach***	**total exposure in *dynamic transport approach***	**transport exposure in *dynamic transport approach***
			**NO_2**		
Total	2.22E+11	2.51E+11	2.50E+11	100%	-
Home		1.52E+11	1.52E+11	61%	-
Work		4.39E+10	4.39E+10	17%	-
Other		2.96E+10	2.96E+10	12%	-
Transport		2.52E+10	2.42E+10	10%	100%
Walking			6.18E+09	2.5%	26%
Bicycles			3.67E+09	1.5%	15%
Motorcars			9.75E+09	3.9%	40%
Buses			2.14E+09	0.9%	9%
Subway trains			1.22E+09	0.5%	5%
Suburban trains			9.91E+08	0.4%	4%
Regional trains			2.88E+08	0.1%	1%
			**$PM_{2.5}$**		
Total	8.71E+10	1.05E+11	1.03E+11	100%	-
Home		5.96E+10	5.96E+10	58%	-
Work		1.66E+10	1.66E+10	16%	-
Other		1.69E+10	1.69E+10	16%	-
Transport		1.20E+10	1.04E+10	10%	100%
Walking			3.11E+09	3%	30%
Bicycles			1.77E+09	1.7%	17%
Motorcars			3.41E+09	3.3%	33%
Buses			9.04E+08	0.9%	9%
Subway Train			6.03E+08	0.6%	6%
Suburban train			4.60E+08	0.4%	4%
Regional train			9.97E+07	0.1%	1%

S6.1 Spatiotemporal variability of simulated total exposure

Besides spatial differences, there are differences in terms of diurnal variability in total exposure. Figure 10 shows the diurnal variation of hourly total exposure averaged over one year for the *dynamic transport* approach versus the *static* and *dynamic approaches* in selected areas. While the difference between the static and dynamic approaches is significant, the differences in total exposure between the dynamic approaches is very small. Nevertheless, in both cases the differences are highest during periods of high transport activity in the morning and evening rush hours. During the night, most people are at home and the emissions by traffic go down, leading to less concentrations and activity. Thus, the exposure calculation is strongly dependent on accurate time profiles for temporal distribution of emissions and in modeling of population dynamics.

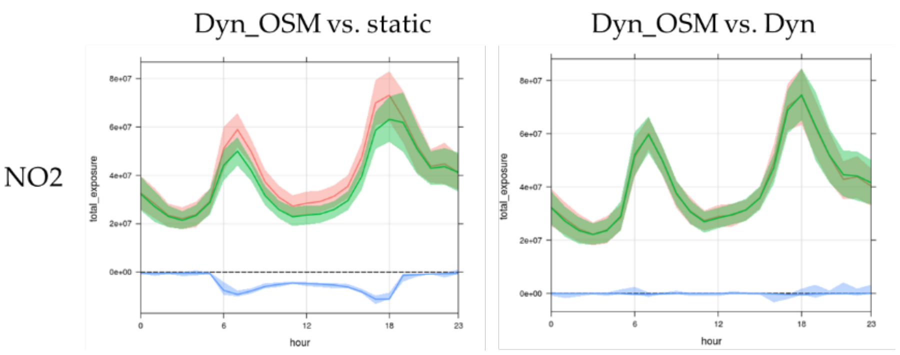

Figure S6-1: Diurnal variation of hourly total exposure to NO_2 averaged over all hourly values for 2016 in the Hamburg area. (**a**) Shows differences between the *dynamic transport approach* and the *static approach*, (**b**) shows differences between the *dynamic transport approach* and the *dynamic approach.*

Supplement S7—Sensitivity of modeled NO_2 and $PM_{2.5}$ exposure

To investigate on uncertainties due to simulated pollutant concentrations in this study, we compared simulated NO_2 and $PM_{2.5}$ concentrations with measurements (section 3.1). The results show highest underestimations at measurement stations close to roads with NMB of up to -35% for NO_2 and −20% for $PM_{2.5}$. To quantify the impact of this underestimation on uncertainty in exposure estimates, we compared the reference emission scenario as defined in section 3.1, with the original lower traffic emissions inventory (minimum scenario). Additionally we created a maximum emissions scenario by scaling the reference scenario with a factor derived from maximum calculated annual NMB. Thus, we applied a factor of 1.4 for NO_2 and a factor of 1.3 for $PM_{2.5}$ to the total road traffic emissions inventory to create a maximum emissions scenario. We applied the minimum and maximum scenarios in our modeling chain to identify minimum and maximum exposure compared to the reference exposure to NO_2 and $PM_{2.5}$ in all microenvironments. The results of the maximum and minimum emissions sensitivity runs (Figure S7-1), reveal relative changes in total exposure and PWE to NO_2 of −9% and +6% for all approaches, and of −1% and +1% for $PM_{2.5}$ respectively. In the transport environments of the *dynamic transport approach* there are uncertainties of −12% to +7% for approach in terms of NO_2 and −2% to +1% for $PM_{2.5}$. Thus, the calculated exposure values show in general a higher sensitivity to changing local NO_2 emissions from road transport, while $PM_{2.5}$ exposure is less sensitive. Nevertheless, for $PM_{2.5}$ the PWE in the *dynamic transport approach* environments Walking, and Buses might rise to values close to or above the WHO AQG limit value for annual $PM_{2.5}$ concentrations of 10 ug/m.

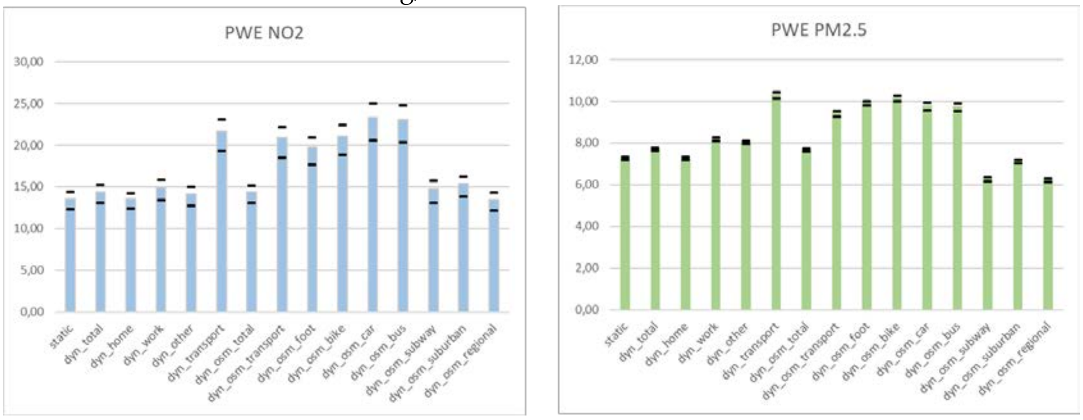

Figure S7-1: Calculated PWE to NO_2 and $PM_{2.5}$ as well as uncertainties in all environments and approaches due to a minimum and a maximum road traffic emission scenario.

Table S7-1: Applied infiltration factors for transport environments in the dynamic_OSM approach and their impacts on total exposure as well as PWE to NO_2 and $PM_{2.5}$ calculations.

		F_{inf}		Exposure change NO_2/$PM_{2.5}$	
**NO_2**	**Min**	**Ref**	**Max**	**Min**	**Max**
Walking	1	1	1	0%	0%
Bicycles	1	1	1	0%	0%
Motorcars	0.7	0.9	1	−22%	11%
Buses	0.7	0.9	1	−22%	11%
Subway trains	0.4	0.6	0.8	−33%	33%
Suburban trains	0.5	0.7	0.9	−29%	29%
Regional trains	0.4	0.6	0.8	−33%	33%
Transport	-	-	-	−14%	+8%
Total	-	-	-	−1%	+1%
**$PM_{2.5}$**	**Min**	**Ref**	**Max**	**Min**	**Max**
Walking	1	1	1	0%	0%
Bicycles	1	1	1	0%	0%
Motorcars	0.5/0.6	0.7/0.8	0.9/1	−27%	+27%
Buses	0.7	0.9	1	−22%	+11%
Subway trains	0.5	0.7	0.9	−29%	+29%

Suburban trains	0.5	0.7	0.8	−29%	+29%
Regional trains	0.4	0.6	0.8	−33%	+33%
Transport	-	-	-	−14%	+13%
Total	-	-	-	−1%	+1%

The calculated impacts of minimum and maximum Finf in different showed linear de- and increases in calculated exposure values. The impact on exposure averaged over all modes of transport is 14% to +8% for both, NO_2 and $PM_{2.5}$. In terms of impact on total exposure in the *dynamic transport approach* the impact is +/−1%. Thus, when analyzing each mode of transport separately, the impact of Finf is highly sensitive. Moreover, the maximum range of uncertainty in the *dynamic transport approach* environments Motorcars and Buses is exceeding the annual WHO AQG limit value of 10 ug/m^3 for $PM_{2.5}$. When analyzing the total exposure of all environments, the impact of Finf for different modes of transport is low due to the relative high contribution of other microenvironments to the total exposure. Nevertheless, in future studies it is desirable to apply Finf for different indoor and transport environments, which represent city-specific building infrastructure and different air-intake or ventilation techniques in buildings, car cabins or inside of buses and trains. This will increase the robustness and benefit of exposure estimates in general and in different transport environments.

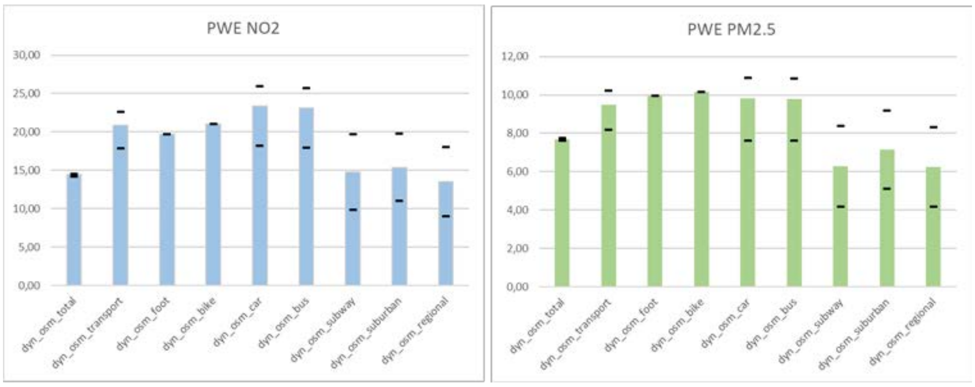

Figure S7-2: Calculated PWE to NO_2 and $PM_{2.5}$ as well as uncertainties in all environments and approaches due to a minimum and a maximum road traffic emission scenario.

S8 Population weighted exposures

Figure S8-1 shows scatter plots of annual total NO_2 and $PM_{2.5}$ exposure grid cell values plotted against the grid cell values of annually averaged NO_2 and $PM_{2.5}$ concentrations for all approaches. This allows for a combined analysis of PWE and concentrations, revealing values with high concentrations but low exposure. It becomes evident, that for both pollutants the PWE is less affected by high concentration values than the dynamic approaches. This leads to higher and more PWE concentrations and finally to more exceedances of the annual limit values. While in the *static approach* the limit value of 40 µg/m³ is exceeded in 190 grid cells, in the *dynamic approach* there 483 and in the *dynamic transport approach* there are 427 exceedances modelled. For PWE $PM_{2.5}$ there are 6 exceedances of the EU limit value of 25 µg/m³ for the static approach, 99 for the *dynamic approach* and 86 for the *dynamic transport approach*, respectively. Looking at the WHO annual limit value of 10 µg/m³ $PM_{2.5}$, we modelled 663 exceedances for the *static approach*, 3184 for the *dynamic approach* and 2631 for the *dynamic transport approach*. Thus, the severity of limit value exceedances is much more evident in both dynamic approaches.

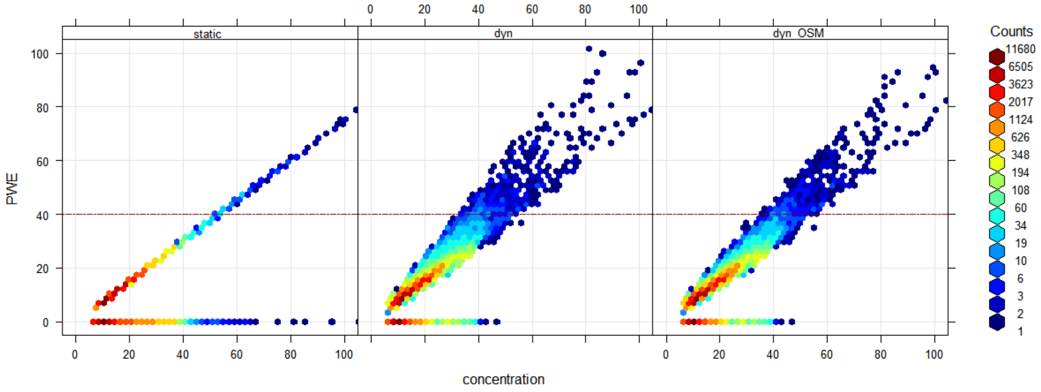

(a)

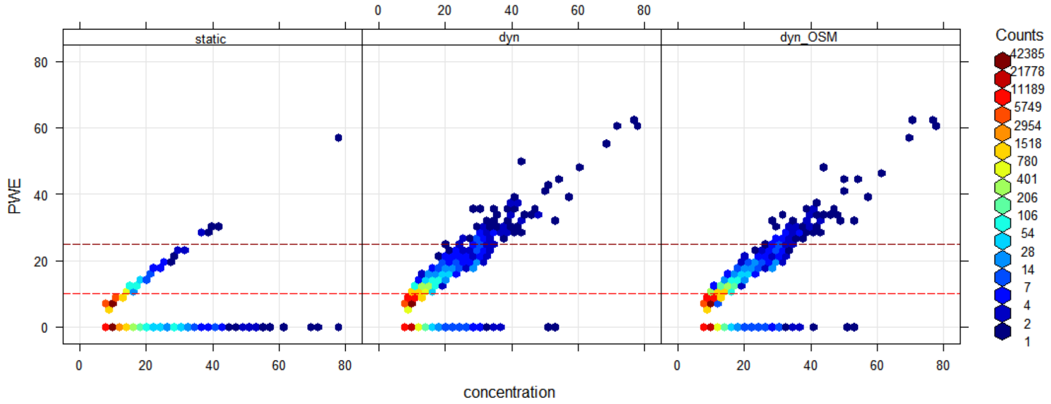

(b)

Figure S8-1: Simulated concentrations versus PWE grid cell values of NO_2 (a) and $PM_{2.5}$ (b). The color code shows counts for pairs of value ranges.

To analyze the impact of road transport to simulated PWE values, we show the levels of relative traffic contribution to each PWE grid cell value in Figure S8-2. For NO_2 higher concentrations and PWE are mostly going along with higher contributions of traffic, which can reach up to 96% and generally influence the exceedances of limit values by a minimum of 50%. For $PM_{2.5}$, the impact of road traffic related PWE can reach a maximum of 77% but is generally lower, with average contributions 8%. Nevertheless, exceedances of the WHO limit value can be influenced by 10–70% (30% in average) of road traffic contribution. Besides some discordant values, the EU limit value exceedances of $PM_{2.5}$ PWE are only affected by a maximum of 10% road traffic contribution. Highest contributions for $PM_{2.5}$ are mostly by industrial point sources at specific locations, which congregate with regional background concentrations.

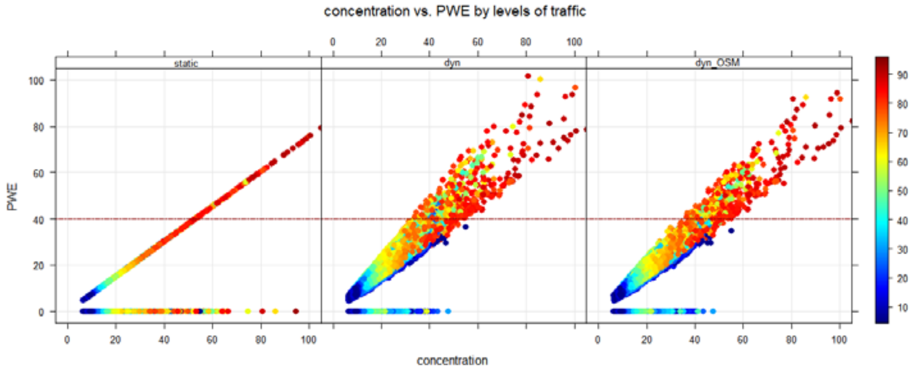

(a)

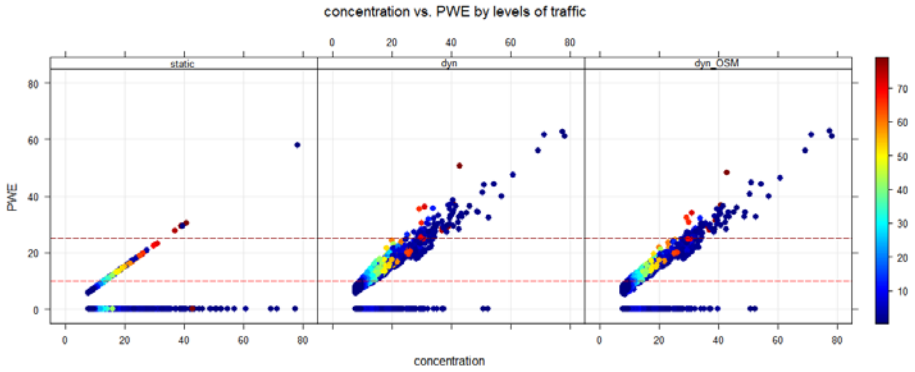

(b)

Figure S8-2: Simulated concentrations and PWE grid cell values of NO_2 (a) and $PM_{2.5}$ (b). The color code shows the relative contribution of road traffic related PWE [%].

Publication bibliography

Ahas, Rein; Silm, Siiri; Järv, Olle; Saluveer, Erki; Tiru, Margus (2010): Using Mobile Positioning Data to Model Locations Meaningful to Users of Mobile Phones. In *Journal of Urban Technology* 17 (1), pp. 3–27. DOI: 10.1080/10630731003597306.

Argyropoulos, Christos D.; Hassan, Hala; Kumar, Prashant; Kakosimos, Konstantinos E. (2020): Measurements and modelling of particulate matter building ingress during a severe dust storm event. In *Building and Environment* 167, p. 106441. DOI: 10.1016/j.buildenv.2019.106441.

Bae, Hyunjoo; Yang, Wonho; Chung, Moonho (2004): Indoor and outdoor concentrations of RSP, NO2 and selected volatile organic compounds at 32 shoe stalls located near busy roadways in Seoul, Korea. In *The Science of the total environment* 323 (1-3), pp. 99–105. DOI: 10.1016/j.scitotenv.2003.09.033.

Baek, Sung-Ok; Kim, Yoon-Shin; Perry, Roger (1997): Indoor air quality in homes, offices and restaurants in Korean urban areas—indoor/outdoor relationships. In *Atmospheric Environment* 31 (4), pp. 529–544. DOI: 10.1016/S1352-2310(96)00215-4.

Baklanov, A.; Hänninen, O.; Slørdal, L. H.; Kukkonen, J.; Bjergene, N.; Fay, B. et al. (2007): Integrated systems for forecasting urban meteorology, air pollution and population exposure. In *Atmos. Chem. Phys.* 7 (3), pp. 855–874. DOI: 10.5194/acp-7-855-2007.

Batista e Silva, Filipe; Poelman, Hugo (2016): Mapping population density in Functional Urban Areas - A method to downscale population statistics to Urban Atlas polygons. JRC Technical Report no. EUR 28194 EN. European Commission Joint Research Centre. Luxembourg.

Beckx, Carolien; Int Panis, Luc; Arentze, Theo; Janssens, Davy; Torfs, Rudi; Broekx, Steven; Wets, Geert (2009a): A dynamic activity-based population modelling approach to evaluate exposure to air pollution. Methods and application to a Dutch urban area. In *Environmental Impact Assessment Review* 29 (3), pp. 179–185. DOI: 10.1016/j.eiar.2008.10.001.

Beckx, Carolien; Int Panis, Luc; Uljee, Inge; Arentze, Theo; Janssens, Davy; Wets, Geert (2009b): Disaggregation of nation-wide dynamic population exposure estimates in The Netherlands: Applications of activity-based transport models. In *Atmospheric Environment* 43 (34), pp. 5454–5462. DOI: 10.1016/j.atmosenv.2009.07.035.

Beekmann, M.; Prévôt, A. S. H.; Drewnick, F.; Sciare, J.; Pandis, S. N.; van der Denier Gon, H. A. C. et al. (2015): In situ, satellite measurement and model evidence on the dominant regional contribution to fine particulate matter levels in the Paris megacity. In *Atmos. Chem. Phys.* 15 (16), pp. 9577–9591. DOI: 10.5194/acp-15-9577-2015.

Beelen, Rob; Hoek, Gerard; Vienneau, Danielle; Eeftens, Marloes; Dimakopoulou, Konstantina; Pedeli, Xanthi et al. (2013): Development of NO2 and NOx land use regression models for estimating air pollution exposure in 36 study areas in Europe – The ESCAPE project. In *Atmospheric Environment* 72, pp. 10–23. DOI: 10.1016/j.atmosenv.2013.02.037.

Berkowicz, R.; Hertel, O.; Larsen, S. E.; Sorensen, N. N.; Nielsen, M. (1997): Modelling traffic pollution in streets. Ministry of Environment and Energy. Roskilde, Denmark. Available online at https://www2.dmu.dk/1_viden/2_Miljoe-tilstand/3_luft/4_spredningsmodeller/5_OSPM/5_description/ModellingTrafficPollution_report.pdf, checked on 1/23/2019.

Bieser, J.; Aulinger, A.; Matthias, V.; Quante, M.; Builtjes, P. (2010): SMOKE for Europe – adaptation, modification and evaluation of a comprehensive emission model for Europe. In *Geosci. Model Dev. Discuss.* 3 (3), pp. 949–1007. DOI: 10.5194/gmdd-3-949-2010.

Bieser, Johannes; Ramacher, Martin Otto Paul; Prank, Marje; Solazzo, Efisio; Uppstu, Andreas (2020): Multi Model Study on the Impact of Emissions on CTMs. In Clemens Mensink, Wanmin Gong, Amir Hakami (Eds.): Air Pollution Modeling and its Application XXVI, vol. 17. Cham: Springer International Publishing (Springer Proceedings in Complexity), pp. 309–315.

Blondeau, P.; Iordache, V.; Poupard, O.; Genin, D.; Allard, F. (2005): Relationship between outdoor and indoor air quality in eight French schools. In *Indoor air* 15 (1), pp. 2–12. DOI: 10.1111/j.1600-0668.2004.00263.x.

Borrego, C.; Sá, E.; Monteiro, A.; Ferreira, J.; Miranda, A. I. (2009): Forecasting human exposure to atmospheric pollutants in Portugal – A modelling approach. In *Atmospheric Environment* 43 (36), pp. 5796–5806. DOI: 10.1016/j.atmosenv.2009.07.049.

Borrego, C.; TCHEPEL, O.; COSTA, A.; MARTINS, H.; Ferreira, J.; MIRANDA, A. (2006): Traffic-related particulate air pollution exposure in urban areas. In *Atmospheric Environment* 40 (37), pp. 7205–7214. DOI: 10.1016/j.atmosenv.2006.06.020.

Bowatte, Gayan; Erbas, Bircan; Lodge, Caroline J.; Knibbs, Luke D.; Gurrin, Lyle C.; Marks, Guy B. et al. (2017): Traffic-related air pollution exposure over a 5-year period is associated with increased risk of asthma and poor lung function in middle age. In *The European respiratory journal* 50 (4). DOI: 10.1183/13993003.02357-2016.

Bravo, Mercedes A.; Fuentes, Montserrat; Zhang, Yang; Burr, Michael J.; Bell, Michelle L. (2012): Comparison of exposure estimation methods for air pollutants: ambient monitoring data and regional air quality simulation. In *Environmental research* 116, pp. 1–10. DOI: 10.1016/j.envres.2012.04.008.

Carslaw, David C.; Ropkins, Karl (2012): openair — An R package for air quality data analysis. In *Environmental Modelling & Software* 27-28, pp. 52–61. DOI: 10.1016/j.envsoft.2011.09.008.

Cepeda, Magda; Schoufour, Josje; Freak-Poli, Rosanne; Koolhaas, Chantal M.; Dhana, Klodian; Bramer, Wichor M.; Franco, Oscar H. (2017): Levels of ambient air pollution according to mode of transport: a systematic review. In *The Lancet Public Health* 2 (1), e23-e34. DOI: 10.1016/S2468-2667(16)30021-4.

Challoner, Avril; Gill, Laurence (2014): Indoor/outdoor air pollution relationships in ten commercial buildings: PM2.5 and NO2. In *Building and Environment* 80, pp. 159–173. DOI: 10.1016/j.buildenv.2014.05.032.

Chen, Chun; Zhao, Bin (2011): Review of relationship between indoor and outdoor particles: I/O ratio, infiltration factor and penetration factor. In *Atmospheric Environment* 45 (2), pp. 275–288. DOI: 10.1016/j.atmosenv.2010.09.048.

Copernicus Land Monitoring Service (2016): Urban Atlas Mapping Guide v4.7. Edited by European Union (EU). Available online at https://land.copernicus.eu/user-corner/technical-library/urban-atlas-2012-mapping-guide-new, updated on 1/13/2019.

Copernicus Land Monitoring Service (2018): Corine Land Cover. Available online at https://land.copernicus.eu/pan-european/corine-land-cover/clc2018, updated on 1/23/2019.

Curtis, Luke; Rea, William; Smith-Willis, Patricia; Fenyves, Ervin; Pan, Yaqin (2006): Adverse health effects of outdoor air pollutants. In *Environment international* 32 (6), pp. 815–830. DOI: 10.1016/j.envint.2006.03.012.

Dewulf, Bart; Neutens, Tijs; Lefebvre, Wouter; Seynaeve, Gerdy; Vanpoucke, Charlotte; Beckx, Carolien; van de Weghe, Nico (2016): Dynamic assessment of exposure to air pollution using

mobile phone data. In *International journal of health geographics* 15, pp. 1–14. DOI: 10.1186/s12942-016-0042-z.

Dhondt, Stijn; Beckx, Carolien; Degraeuwe, Bart; Lefebvre, Wouter; Kochan, Bruno; Bellemans, Tom et al. (2012): Health impact assessment of air pollution using a dynamic exposure profile: Implications for exposure and health impact estimates. In *Environmental Impact Assessment Review* 36, pp. 42–51. DOI: 10.1016/j.eiar.2012.03.004.

Dias, Daniela; Tchepel, Oxana (2018): Spatial and Temporal Dynamics in Air Pollution Exposure Assessment. In *International journal of environmental research and public health* 15 (3). DOI: 10.3390/ijerph15030558.

Dionisio, Kathie L.; Baxter, Lisa K.; Burke, Janet; Özkaynak, Halûk (2016): The importance of the exposure metric in air pollution epidemiology studies: When does it matter, and why? In *Air Qual Atmos Health* 9 (5), pp. 495–502. DOI: 10.1007/s11869-015-0356-1.

Dons, Evi; Int Panis, Luc; van Poppel, Martine; Theunis, Jan; Willems, Hanny; Torfs, Rudi; Wets, Geert (2011): Impact of time–activity patterns on personal exposure to black carbon. In *Atmospheric Environment* 45 (21), pp. 3594–3602. DOI: 10.1016/j.atmosenv.2011.03.064.

Elessa Etuman, Arthur; Coll, Isabelle (2018): OLYMPUS v1.0: development of an integrated air pollutant and GHG urban emissions model – methodology and calibration over greater Paris. In *Geosci. Model Dev.* 11 (12), pp. 5085–5111. DOI: 10.5194/gmd-11-5085-2018.

European Environment Agency (2019): Air quality in Europe. 2019 report. Luxembourg: Publications Office of the European Union (EEA report, No 10/2019).

European Environment Agency (2020): Health impacts of air pollution. Available online at https://www.eea.europa.eu/themes/air/health-impacts-of-air-pollution, updated on 1/28/2020, checked on 2/16/2020.

Eurostat (2020): Urban population exposure to air pollution by particulate matter. Available online at https://ec.europa.eu/eurostat/web/products-datasets/-/T2020_RN210, updated on 2/21/2020, checked on 2/21/2020.

Florczyk, A. J.; Cobane, C.; Ehrlich, D.; Freire, S.; Kemper, T.; Maffeini, L. et al. (2019): GHSL Data Package 2019. JRC Technical Report. EUR 29788 EN. Edited by Publications Office of the European Union. Luxembourg.

Fridell, Erik; Haeger-Eugensson, Marie; Moldanova, Jana; Forsberg, Bertil; Sjöberg, Karin (2014): A modelling study of the impact on air quality and health due to the emissions from E85 and petrol fuelled cars in Sweden. In *Atmospheric Environment* 82, pp. 1–8. DOI: 10.1016/j.atmosenv.2013.10.002.

Fujita, Eric M.; Campbell, David E.; Arnott, W. Patrick; Johnson, Ted; Ollison, Will (2014): Concentrations of mobile source air pollutants in urban microenvironments. In *Journal of the Air & Waste Management Association (1995)* 64 (7), pp. 743–758. DOI: 10.1080/10962247.2013.872708.

Gariazzo, Claudio; Pelliccioni, Armando; Bolignano, Andrea (2016): A dynamic urban air pollution population exposure assessment study using model and population density data derived by mobile phone traffic. In *Atmospheric Environment* 131, pp. 289–300. DOI: 10.1016/j.atmosenv.2016.02.011.

Gately, Conor K.; Hutyra, Lucy R.; Peterson, Scott; Sue Wing, Ian (2017): Urban emissions hotspots. Quantifying vehicle congestion and air pollution using mobile phone GPS data. In *Environmental pollution (Barking, Essex : 1987)* 229, pp. 496–504. DOI: 10.1016/j.envpol.2017.05.091.

Gerharz, Lydia E.; Klemm, Otto; Broich, Anna V.; Pebesma, Edzer (2013): Spatio-temporal modelling of individual exposure to air pollution and its uncertainty. In *Atmospheric Environment* 64, pp. 56–65. DOI: 10.1016/j.atmosenv.2012.09.069.

Granier, C.; Darras, S.; Denier van der Gon, H.; Doubalova, J.; Elguindi, N.; Galle, B. et al. (2019): The Copernicus Atmosphere Monitoring Service global and regional emissions (April 2019 version). Available online at https://atmosphere.copernicus.eu/sites/default/files/2019-06/cams_emissions_general_document_apr2019_v7.pdf, checked on 2/6/2020.

Hamer, Paul D.; Walker, Sam-Erik; Sousa-Santos, Gabriela; Vogt, Matthias; Vo-Thanh, Dam; Lopez-Aparicio, Susana et al. (2019): The urban dispersion model EPISODE. Part 1: A Eulerian and subgrid-scale air quality model and its application in Nordic winter conditions. In *Geosci. Model Dev. Discuss.*, pp. 1–57. DOI: 10.5194/gmd-2019-199.

Hamra, Ghassan B.; Laden, Francine; Cohen, Aaron J.; Raaschou-Nielsen, Ole; Brauer, Michael; Loomis, Dana (2015): Lung Cancer and Exposure to Nitrogen Dioxide and Traffic. A Systematic Review and Meta-Analysis. In *Environmental health perspectives* 123 (11), pp. 1107–1112. DOI: 10.1289/ehp.1408882.

Hanna, Steven; Chang, Joseph (2012): Acceptance criteria for urban dispersion model evaluation. In *Meteorol Atmos Phys* 116 (3-4), pp. 133–146. DOI: 10.1007/s00703-011-0177-1.

Hänninen, Otto O.; Alm, Sari; Katsouyanni, Klea; Künzli, Nino; Maroni, Marco; Nieuwenhuijsen, Mark J. et al. (2004): The EXPOLIS study: implications for exposure research and environmental policy in Europe. In *Journal of exposure analysis and environmental epidemiology* 14 (6), pp. 440–456. DOI: 10.1038/sj.jea.7500342.

Hazlehurst, Marnie F.; Spalt, Elizabeth W.; Nicholas, Tyler P.; Curl, Cynthia L.; Davey, Mark E.; Burke, Gregory L. et al. (2018): Contribution of the in-vehicle microenvironment to individual ambient-source nitrogen dioxide exposure: the Multi-Ethnic Study of Atherosclerosis and Air Pollution. In *Journal of exposure science & environmental epidemiology* 28 (4), pp. 371–380. DOI: 10.1038/s41370-018-0025-1.

Heroux, M. E.; Braubach, M.; Korol, N.; Krzyzanowski, M.; Paunovic, E.; Zastenskaya, I. (2013): The main conclusions about the medical aspects of air pollution. The projects REVIHAAP and HRAPIE WHO/EC. In *Gigiena i sanitariia* (6), pp. 9–14.

Hoffmann, Peter; Fischereit, Jana; Heitmann, Stefan; Schlünzen, K.; Gasser, Ingenuin (2018): Modeling Exposure to Heat Stress with a Simple Urban Model. In *Urban Science* 2 (1), p. 9. DOI: 10.3390/urbansci2010009.

Holtermann, Linus; Alkis, Otto; Schulze, Sven (2013): Pendeln in Hamburg. HWWI Policy Paper 83. Edited by Hamburgisches WeltWirtschaftsInstitut (HWWI). Hamburg. Available online at http://www.hwwi.org/uploads/tx_wilpubdb/HWWI-Policy_Paper_83.pdf, checked on 2/2/2020.

Horsdal, Henriette Thisted; Agerbo, Esben; McGrath, John Joseph; Vilhjálmsson, Bjarni Jóhann; Antonsen, Sussie; Closter, Ane Marie et al. (2019): Association of Childhood Exposure to Nitrogen Dioxide and Polygenic Risk Score for Schizophrenia With the Risk of Developing Schizophrenia. In *JAMA network open* 2 (11), e1914401. DOI: 10.1001/jamanetworkopen.2019.14401.

Hurley, Peter J.; Physick, William L.; Luhar, Ashok K. (2005): TAPM. A practical approach to prognostic meteorological and air pollution modelling. In *Environmental Modelling & Software* 20 (6), pp. 737–752. DOI: 10.1016/j.envsoft.2004.04.006.

Hurley, Peter John (2008): TAPM. Technical description. Aspendale, Vic.: CSIRO (CSIRO Marine and Atmospheric Research paper, 025).

Hvidtfeldt, Ulla Arthur; Geels, Camilla; Sørensen, Mette; Ketzel, Matthias; Khan, Jibran; Tjønneland, Anne et al. (2019): Long-term residential exposure to PM2.5 constituents and mortality in a Danish cohort. In *Environment international* 133 (Pt B), p. 105268. DOI: 10.1016/j.envint.2019.105268.

Ibarra-Espinosa, Sergio; Ynoue, Rita; O' Sullivan, Shane; Pebesma, Edzer; Andrade, María de Fátima; Osses, Mauricio (2018): VEIN v0.2.2: an R package for bottom–up vehicular emissions inventories. In *Geosci. Model Dev.* 11 (6), pp. 2209–2229. DOI: 10.5194/gmd-11-2209-2018.

Im, Ulas; Brandt, Jørgen; Geels, Camilla; Hansen, Kaj Mantzius; Christensen, Jesper Heile; Andersen, Mikael Skou et al. (2018): Assessment and economic valuation of air pollution impacts on human health over Europe and the United States as calculated by a multi-model ensemble in the framework of AQMEII3. In *Atmos. Chem. Phys.* 18 (8), pp. 5967–5989. DOI: 10.5194/acp-18-5967-2018.

Jerrett, Michael; Arain, Altaf; Kanaroglou, Pavlos; Beckerman, Bernardo; Potoglou, Dimitri; Sahsuvaroglu, Talar et al. (2005): A review and evaluation of intraurban air pollution exposure models. In *Journal of exposure analysis and environmental epidemiology* 15 (2), pp. 185–204. DOI: 10.1038/sj.jea.7500388.

Jia, Xu; Yang, Xuan; Hu, Dayu; Dong, Wei; Yang, Fan; Liu, Qi et al. (2018): Short-term effects of particulate matter in metro cabin on heart rate variability in young healthy adults: Impacts of particle size and source. In *Environmental research* 167, pp. 292–298. DOI: 10.1016/j.envres.2018.07.017.

Jiao, Wan; Frey, H. Christopher (2013): Method for Measuring the Ratio of In-Vehicle to Near-Vehicle Exposure Concentrations of Airborne Fine Particles. In *Transportation Research Record* 2341 (1), pp. 34–42. DOI: 10.3141/2341-04.

Karl, Matthias; Walker, Sam-Erik; Solberg, Sverre; Ramacher, Martin O. P. (2019): The Eulerian urban dispersion model EPISODE – Part 2: Extensions to the source dispersion and photochemistry for EPISODE–CityChem v1.2 and its application to the city of Hamburg. In *Geosci. Model Dev.* 12 (8), pp. 3357–3399. DOI: 10.5194/gmd-12-3357-2019.

Kousa, Anu; Kukkonen, Jaakko; Karppinen, Ari; Aarnio, Päivi; Koskentalo, Tarja (2002): A model for evaluating the population exposure to ambient air pollution in an urban area. In *Atmospheric Environment* 36 (13), pp. 2109–2119. DOI: 10.1016/S1352-2310(02)00228-5.

Kuenen, J. J. P.; Visschedijk, A. J. H.; Jozwicka, M.; van der Denier Gon, H. A. C. (2014): TNO-MACC_II emission inventory; a multi-year (2003–2009) consistent high-resolution European emission inventory for air quality modelling. In *Atmos. Chem. Phys.* 14 (20), pp. 10963–10976. DOI: 10.5194/acp-14-10963-2014.

Kuik, Friderike; Kerschbaumer, Andreas; Lauer, Axel; Lupascu, Aurelia; Schneidemesser, Erika von; Butler, Tim M. (2018): Top–down quantification of NO_x emissions from traffic in an urban area using a high-resolution regional atmospheric chemistry model. In *Atmos. Chem. Phys.* 18 (11), pp. 8203–8225. DOI: 10.5194/acp-18-8203-2018.

Künzli, N.; Kaiser, R.; Medina, S.; Studnicka, M.; Chanel, O.; Filliger, P. et al. (2000): Public-health impact of outdoor and traffic-related air pollution: a European assessment. In *The Lancet* 356 (9232), pp. 795–801. DOI: 10.1016/S0140-6736(00)02653-2.

Latza, Ute; Gerdes, Silke; Baur, Xaver (2009): Effects of nitrogen dioxide on human health: systematic review of experimental and epidemiological studies conducted between 2002 and 2006. In *International journal of hygiene and environmental health* 212 (3), pp. 271–287. DOI: 10.1016/j.ijheh.2008.06.003.

Lepeule, Johanna; Laden, Francine; Dockery, Douglas; Schwartz, Joel (2012): Chronic exposure to fine particles and mortality: an extended follow-up of the Harvard Six Cities study from 1974 to 2009. In *Environmental health perspectives* 120 (7), pp. 965–970. DOI: 10.1289/ehp.1104660.

Li, Zhiyuan; Che, Wenwei; Frey, H. Christopher; Lau, Alexis K. H. (2018): Factors affecting variability in PM2.5 exposure concentrations in a metro system. In *Environmental research* 160, pp. 20–26. DOI: 10.1016/j.envres.2017.09.006.

Liu, Xiaozhen; Frey, H. Christopher (2011): Modeling Of In-Vehicle Human Exposure to Ambient Fine Particulate Matter. In *Atmospheric Environment* 45 (27), pp. 4745–4752. DOI: 10.1016/j.atmosenv.2011.04.019.

Marécal, V.; Peuch, V.-H.; Andersson, C.; Andersson, S.; Arteta, J.; Beekmann, M. et al. (2015): A regional air quality forecasting system over Europe: the MACC-II daily ensemble production. In *Geosci. Model Dev.* 8 (9), pp. 2777–2813. DOI: 10.5194/gmd-8-2777-2015.

Matthias, Volker; Arndt, Jan A.; Aulinger, Armin; Bieser, Johannes; van der Denier Gon, Hugo; Kranenburg, Richard et al. (2018): Modeling emissions for three-dimensional atmospheric chemistry transport models. In *Journal of the Air & Waste Management Association (1995)* 68 (8), pp. 763–800. DOI: 10.1080/10962247.2018.1424057.

Meier, Reto; Schindler, Christian; Eeftens, Marloes; Aguilera, Inmaculada; Ducret-Stich, Regina E.; Ineichen, Alex et al. (2015): Modeling indoor air pollution of outdoor origin in homes of SAPALDIA subjects in Switzerland. In *Environment international* 82, pp. 85–91. DOI: 10.1016/j.envint.2015.05.013.

Nyhan, Marguerite; Grauwin, Sebastian; Britter, Rex; Misstear, Bruce; McNabola, Aonghus; Laden, Francine et al. (2016): "Exposure Track"-The Impact of Mobile-Device-Based Mobility Patterns on Quantifying Population Exposure to Air Pollution. In *Environmental science & technology* 50 (17), pp. 9671–9681. DOI: 10.1021/acs.est.6b02385.

Onat, Burcu; Stakeeva, Baktygul (2013): Personal exposure of commuters in public transport to PM2.5 and fine particle counts. In *Atmospheric Pollution Research* 4 (3), pp. 329–335. DOI: 10.5094/APR.2013.037.

Ott, Wayne R. (1982): Concepts of human exposure to air pollution. In *Environment international* 7 (3), pp. 179–196. DOI: 10.1016/0160-4120(82)90104-0.

Özkaynak, Halûk; Baxter, Lisa K.; Dionisio, Kathie L.; Burke, Janet (2013): Air pollution exposure prediction approaches used in air pollution epidemiology studies. In *Journal of exposure science & environmental epidemiology* 23 (6), pp. 566–572. DOI: 10.1038/jes.2013.15.

Padgham, Mark; Lovelace, Robin; Salmon, Maëlle; Rudis, Bob (2017): osmdata. In *JOSS* 2 (14), p. 305. DOI: 10.21105/joss.00305.

Picornell, Miguel; Ruiz, Tomás; Borge, Rafael; García-Albertos, Pedro; La Paz, David de; Lumbreras, Julio (2019): Population dynamics based on mobile phone data to improve air pollution exposure assessments. In *Journal of exposure science & environmental epidemiology*, pp. 278–291. DOI: 10.1038/s41370-018-0058-5.

R Core Team (2019): R: A Language and Environment for Statistical Computing. Vienna, Austria. Available online at https://www.R-project.org/.

Ragettli, Martina S.; Tsai, Ming-Yi; Braun-Fahrländer, Charlotte; Nazelle, Audrey de; Schindler, Christian; Ineichen, Alex et al. (2014): Simulation of population-based commuter exposure to NO_2 using different air pollution models. In *International journal of environmental research and public health* 11 (5), pp. 5049–5068. DOI: 10.3390/ijerph110505049.

Ramacher, Martin Otto Paul; Karl, Matthias; Aulinger, Armin; Bieser, Johannes (2020): Population Exposure to Emissions from Industry, Traffic, Shipping and Residential Heating in the Urban Area of Hamburg. In Clemens Mensink, Wanmin Gong, Amir Hakami (Eds.): Air Pollution Modeling and its Application XXVI, vol. 15. Cham: Springer International Publishing (Springer Proceedings in Complexity), pp. 177–183.

Ramacher, Martin Otto Paul; Karl, Matthias; Bieser, Johannes; Jalkanen, Jukka-Pekka; Johansson, Lasse (2019): Urban population exposure to NOx emissions from local shipping in three Baltic Sea harbour cities – a generic approach. In *Atmos. Chem. Phys.* 19 (14), pp. 9153–9179. DOI: 10.5194/acp-19-9153-2019.

Rasche, Marius; Walther, Mario; Schiffner, Rene; Kroegel, Nasim; Rupprecht, Sven; Schlattmann, Peter et al. (2018): Rapid increases in nitrogen oxides are associated with acute myocardial infarction. A case-crossover study. In *European journal of preventive cardiology* 25 (16), pp. 1707–1716. DOI: 10.1177/2047487318755804.

Reis, Stefan; Liška, Tomáš; Vieno, Massimo; Carnell, Edward J.; Beck, Rachel; Clemens, Tom et al. (2018): The influence of residential and workday population mobility on exposure to air pollution in the UK. In *Environment international* 121 (Pt 1), pp. 803–813. DOI: 10.1016/j.envint.2018.10.005.

Rivas, Ioar; Kumar, Prashant; Hagen-Zanker, Alex (2017): Exposure to air pollutants during commuting in London: Are there inequalities among different socio-economic groups? In *Environment international* 101, pp. 143–157. DOI: 10.1016/j.envint.2017.01.019.

Robert J. Hijmans & Jacob van Etten (2012): raster: Geographic analysis and modeling with raster data. Available online at http://CRAN.R-project.org/package=raster.

Salonen, Heidi; Salthammer, Tunga; Morawska, Lidia (2019): Human exposure to NO2 in school and office indoor environments. In *Environment international* 130, p. 104887. DOI: 10.1016/j.envint.2019.05.081.

Shekarrizfard, Maryam; Faghih-Imani, Ahmadreza; Hatzopoulou, Marianne (2016): An examination of population exposure to traffic related air pollution: Comparing spatially and temporally resolved estimates against long-term average exposures at the home location. In *Environmental research* 147, pp. 435–444. DOI: 10.1016/j.envres.2016.02.039.

Shen, Jialei; Gao, Zhi (2019): Commuter exposure to particulate matters in four common transportation modes in Nanjing. In *Building and Environment* 156, pp. 156–170. DOI: 10.1016/j.buildenv.2019.04.018.

Siddika, Nazeeba; Rantala, Aino K.; Antikainen, Harri; Balogun, Hamudat; Amegah, A. Kofi; Ryti, Niilo R. I. et al. (2019): Synergistic effects of prenatal exposure to fine particulate matter (PM2.5) and ozone (O3) on the risk of preterm birth: A population-based cohort study. In *Environmental research* 176, p. 108549. DOI: 10.1016/j.envres.2019.108549.

Simpson, D.; Fagerli, H.; Johnson, J. E.; Tsyro, S.; Wind, P. (2003): Transboundary acidification, eutrophication and ground level ozone in Europe. Part II. Unified EMEP model performance. EMEP status report 1/2003. ISSN 0806-4520. Norwegian Meteorological Institute, Oslo, Norway.

Singh, Vikas; Sokhi, Ranjeet S.; Kukkonen, Jaakko (2020): An approach to predict population exposure to ambient air PM2.5 concentrations and its dependence on population activity for the megacity London. In *Environmental pollution (Barking, Essex : 1987)* 257, p. 113623. DOI: 10.1016/j.envpol.2019.113623.

Smith, James David; Mitsakou, Christina; Kitwiroon, Nutthida; Barratt, Ben M.; Walton, Heather A.; Taylor, Jonathon G. et al. (2016): London Hybrid Exposure Model: Improving Human Exposure

Estimates to NO2 and PM2.5 in an Urban Setting. In *Environmental science & technology* 50 (21), pp. 11760–11768. DOI: 10.1021/acs.est.6b01817.

Soares, J.; Kousa, A.; Kukkonen, J.; Matilainen, L.; Kangas, L.; Kauhaniemi, M. et al. (2014): Refinement of a model for evaluating the population exposure in an urban area. In *Geosci. Model Dev.* 7 (5), pp. 1855–1872. DOI: 10.5194/gmd-7-1855-2014.

Statistisches Amt für Hamburg und Schleswig-Holstein (2019): Statistisches Jahrbuch Hamburg 2018/2019. Hamburg.

Steinle, Susanne; Reis, Stefan; Sabel, Clive Eric (2013): Quantifying human exposure to air pollution--moving from static monitoring to spatio-temporally resolved personal exposure assessment. In *The Science of the total environment* 443, pp. 184–193. DOI: 10.1016/j.scitotenv.2012.10.098.

Tang, Lin; Ramacher, Martin Otto Paul; Moldanova, Jana; Matthias, Volker; Karl, Matthias; Johansson, Lasse (2020): The impact of ship emissions on air quality and human health in the Gothenburg area – Part 1: Current situtation. In *Atmos. Chem. Phys. Discuss.*, in preparation.

Tayarani, Mohammad; Rowangould, Gregory (2019): Estimating exposure to fine particulate matter emissions from vehicle traffic: Exposure misclassification and daily activity patterns in a large, sprawling region. In *Environmental research* 182, p. 108999. DOI: 10.1016/j.envres.2019.108999.

Thunis, P.; Pederzoli, A.; Pernigotti, D. (2012): Performance criteria to evaluate air quality modeling applications. In *Atmospheric Environment* 59, pp. 476–482. DOI: 10.1016/j.atmosenv.2012.05.043.

Tong, Zheming; Li, Yue; Westerdahl, Dane; Adamkiewicz, Gary; Spengler, John D. (2019): Exploring the effects of ventilation practices in mitigating in-vehicle exposure to traffic-related air pollutants in China. In *Environment international* 127, pp. 773–784. DOI: 10.1016/j.envint.2019.03.023.

Wang, Liangzhu Leon; Dols, W. Stuart; Chen, Qingyan (2010): Using CFD Capabilities of CONTAM 3.0 for Simulating Airflow and Contaminant Transport in and around Buildings. In *HVAC&R Res.* 16 (6), pp. 749–763. DOI: 10.1080/10789669.2010.10390932.

WHO (2006): Air Quality Guidelines. Global Update 2005. Particulate matter, ozone, nitrogen dioxide, and sulfur dioxide. Copenhagen, Denmark: World Health Organization.

WHO (2016a): Health risk assessment of air pollution – general principles. Copenhagen, Denmark. Available online at http://www.euro.who.int/__data/assets/pdf_file/0006/298482/Health-risk-assessment-air-pollution-General-principles-en.pdf, checked on 8/19/2019.

WHO (2016b): WHO Expert Consultation: Available evidence for the future update of the WHO Global Air Quality Guidelines (AQGs). Meeting report. Bonn.

WHO (2018): WHO Global Ambient Air Quality Database (update 2018). Available online at https://www.who.int/airpollution/data/cities/en/, checked on 2/21/2020.

Wing, Sam E.; Bandoli, Gretchen; Telesca, Donatello; Su, Jason G.; Ritz, Beate (2018): Chronic exposure to inhaled, traffic-related nitrogen dioxide and a blunted cortisol response in adolescents. In *Environmental research* 163, pp. 201–207. DOI: 10.1016/j.envres.2018.01.011.

Wu, Mei-Yi; Lo, Wei-Cheng; Chao, Chia-Ter; Wu, Mai-Szu; Chiang, Chih-Kang (2020): Association between air pollutants and development of chronic kidney disease: A systematic review and meta-analysis. In *The Science of the total environment* 706, p. 135522. DOI: 10.1016/j.scitotenv.2019.135522.

Yang, Liang; Hoffmann, Peter; Scheffran, Jürgen; Rühe, Sven; Fischereit, Jana; Gasser, Ingenuin (2018): An Agent-Based Modeling Framework for Simulating Human Exposure to Environmental Stresses in Urban Areas. In *Urban Science* 2 (2), p. 36. DOI: 10.3390/urbansci2020036.

Yu, Qi; Lu, Yi; Xiao, Shan; Shen, Junxiu; Li, Xun; Ma, Weichun; Chen, Limin (2012): Commuters' exposure to PM1 by common travel modes in Shanghai. In *Atmospheric Environment* 59, pp. 39–46. DOI: 10.1016/j.atmosenv.2012.06.001.

Zou, Bin; Wilson, J. Gaines; Zhan, F. Benjamin; Zeng, Yongnian (2009): Air pollution exposure assessment methods utilized in epidemiological studies. In *Journal of environmental monitoring : JEM* 11 (3), pp. 475–490. DOI: 10.1039/b813889c.
